# Quantifying Opponent Process Dynamics in Pornography Use and Masturbation: An Exploratory Ecological Momentary Assessment Study

**DOI:** 10.1007/s10508-025-03287-z

**Published:** 2025-11-21

**Authors:** Nathan I. N. Henry, Mangor Pedersen, Matt Williams, Liesje Donkin

**Affiliations:** 1 Department of Psychology and Neuroscience, Auckland University of Technology, Auckland, New Zealand; 2 School of Psychological Science, University of Western Australia, 35 Stirling Highway, Crawley, WA 6009 Australia; 3 School of Psychology, Massey University, Auckland, New Zealand

**Keywords:** Pornography, Problematic pornography use, Opponent process, Depression, Ecological Momentary Assessment

## Abstract

The causal relationships between pornography use, masturbation, moral incongruence, and mental health are poorly understood. While the link between problematic pornography use (PPU) and depression is well documented, the temporal dynamics associated with pornography use and masturbation have not yet been quantified. Utilizing an exploratory ecological momentary assessment (EMA) design, we measured the temporal dynamics of cognitive and affective state variables collected from 22 participants before, during, and after pornography use and masturbation, and examined the moderating role of moral incongruence in these relationships. Participants completed an initial survey followed by a four-week EMA, capturing data on sexual activities and cognitive and affective state variables. Bayesian hierarchical mixed-effects models were employed to analyze temporal dynamics. Findings suggest that pornography use and masturbation were linked to changes in affective and cognitive states that spiked both before and after sexual episodes. These states were generally more pronounced and sustained in participants with high moral incongruence, who experienced transient increases in shame, guilt, difficulty thinking, relationship disconnectedness, craving for sexual intercourse, and mood deterioration after pornography use and masturbation. These shifts typically persisted for several hours before decaying to baseline levels. High-incongruence participants also experienced strong spikes in craving prior to sexual episodes, while low-incongruence participants experienced craving spikes after pornography use and masturbation, indicating possible sensitization effects. Our findings also signalled potential evidence for “brain fog” following pornography use in both low and high-incongruence participants. Further, we discovered opponent process dynamics in the mood of high-incongruence participants, providing a possible causal mechanism that may explain how PPU can lead to depression.

**Supplementary Information:**

The online version contains supplementary material available at 10.1007/s10508-025-03287-z.

## Introduction

### Problematic Pornography Use

Problematic pornography use (PPU) is characterized by a pattern of pornography consumption that causes distress and functional impairment across personal, social, or occupational areas, and is marked by an inability to control pornography use despite these adverse effects (Bőthe et al., 2024a, 2024b; Ince et al., 2021).^1^ The International Sex Survey has estimated that between 3.2 and 16.6% of the populations from 42 countries are predicted to have PPU (Bőthe et al., 2024a, 2024b), which has only recently been clinically recognized as an impulse control disorder, having been added as a subcategory of Compulsive Sexual Behavior Disorder in the 11th edition of the *International Classification of Diseases* (Kraus et al., 2018).

Yet despite this recognition, there remains controversy over whether PPU should be classified as an addiction or an impulse control disorder (Bőthe et al., 2024a, 2024b; Brand et al., 2020; Pistre et al., 2023). While compulsive pornography use can have negative psychosocial consequences, PPU has features overlapping both behavioral addictions and impulse control disorders, and as such may have a distinct aetiology from true addiction (Grubbs et al., 2019a, 2019b; Ravish et al., 2024). Yet there remains a high prevalence of anecdotal evidence for self-perceived “pornography addiction” in popular culture and online discourse (Chasioti & Binnie, 2021). Therefore, resolving the diagnostic status of PPU remains a key priority for both researchers and clinicians.

Prior research has linked PPU to depressed mood, decreased relationship connectedness (Cardoso et al., 2023; Lambert et al., 2012; Maitland & Neilson, 2023; Mestre-Bach & Potenza, 2023; Vaillancourt-Morel et al., 2024), increased anxiety, guilt, shame, loneliness, difficulty thinking, and suicidal thoughts (Cardoso et al., 2023; Fernandez et al., 2021; Guidry et al., 2020; Maitland & Neilson, 2023; McGraw et al., 2024; Mestre-Bach & Potenza, 2023), along with other psychosocial disorders (Chasioti & Binnie, 2021). Despite some evidence that pornography use can have a positive effect on attitudes towards sexuality (McKee, 2007; Rissel et al., 2017), it has also been associated with lower relationship satisfaction and commitment, especially when pornography use occurs without the partner’s consenting knowledge (Bechara et al., 2003; Floyd & Grubbs, 2022; Grubbs et al., 2022a, 2022b; Kohut et al., 2017; Lambert et al., 2012; Perry, 2016; Vaillancourt-Morel et al., 2024; Willoughby & Dover, 2024; Wright et al., 2017; Zitzman & Butler, 2009). The literature also suggests that PPU can significantly affect the user’s relationships with family, friends, and colleagues (Bridges & Morokoff, 2011; Fagan, 2009; Stack et al., 2004; Stewart & Szymanski, 2012). Hence, there are concerns over the societal and public health consequences of increasing pornographic consumption, regardless of its diagnostic status (Grubbs & Perry, 2019; Lambert et al., 2012).

It is important to account for masturbation when quantifying the effects of pornography use, as the two behaviors often co-occur but may also occur independently, with potentially distinct psychological and relational outcomes (Perry, 2020; Prause, 2017, 2019). The context in which masturbation occurs likely moderates its impact on mental health. For example, Perry (2020) found that once masturbation frequency was controlled for, the association between pornography use and both depression symptoms and decreased relationship satisfaction was reduced. Thus, differentiating these behaviors may help to clarify whether observed cognitive and emotional effects arise from visual stimulation, physical sexual release, or the interaction of these factors.

Yet behavioral mechanisms alone do not fully account for pornography-related distress. Recently, moral incongruence—defined as the internal conflict or distress that arises when an individual’s behavior violates their own moral, ethical, or religious values—has emerged as a key psychological factor in PPU (Grubbs & Perry, 2019; Grubbs et al., 2019a, 2019b, 2022a, 2022b; Lewczuk et al., 2020; Perry, 2018). A review by Floyd and Grubbs (2022) identified that moral incongruence regarding one’s own pornography use serves as a moderating factor for various pornography-related issues, such as emotional distress, relationship difficulties, and the sensation of addiction. Moral incongruence is influenced by multiple factors, including an individual’s religious beliefs, cultural norms, conservative sexual values, apprehensions about sexual exploitation, and the potential impact on interpersonal relationships (Chen et al., 2023; Hoagland et al., 2023; Ince et al., 2023; Lewczuk et al., 2021; Su et al., 2024).

However, a glaring issue remains: the psychological mechanisms linking pornography use to negative mental health outcomes have not yet been confirmed or quantified. While a substantial body of correlational research suggests such associations, and despite a growing number of longitudinal studies in this area (e.g., see Bőthe et al., 2022; Chen et al., 2022; Dawson et al., 2019; Kohut & Štulhofer, 2018; Leonhardt & Willoughby, 2018; Mattebo et al., 2018; McGraw et al., 2024; Milas et al., 2020; Muusses et al., 2015; Perry, 2017; Perry & Davis, 2017; Šević et al., 2020; Wright, 2012, 2013, 2023), few of these studies offer the temporal resolution needed to capture the affective and cognitive dynamics associated with pornography use and masturbation on a momentary basis. Some recent work has begun to address this limitation using experience sampling methods (Bőthe et al., 2020, 2021a, 2021b; Stark et al., 2024; Vaillancourt-Morel et al., 2020, 2024; Wordecha et al., 2018), but the field still lacks the necessary granularity to determine directionality—whether pornography use causes the associated mental health issues, results from them, or if the relationship is bidirectional. Without such data, conclusions about the causal impact of pornography on mental health remain speculative.

In summary, the causal relationship between moral incongruence, pornography use, and mental health remains unclear. Understanding the neuropsychological and behavioral mechanisms that drive the temporal variations in affective and cognitive dynamics around pornography use and masturbation is vital to clarify whether PPU is merely an impulse control disorder, or a true addiction that has elements of impulsivity. It will also help to enhance identification of clinically relevant thresholds for PPU.

### Affective Chronometry

Many neuropsychological phenomena have a curvilinear decay, such as an exponential or power-law decrease, in response to a stimulus. Examples that unfold over several hours include memory retention (Candia et al., 2019; Kahana & Adler, 2017; Murre & Dros, 2015; Peterson & Peterson, 1959; Subirana et al., 2017; Wixted & Ebbesen, 1991) and emotional responses (Anderson & Adolphs, 2014; Botelho et al., 2018; Dejonckheere et al., 2020; Feidakis, 2011; Heller et al., 2015; Picard, 1997; Solomon & Corbit, 1974). These temporal dynamics can be measured using Ecological Momentary Assessment (EMA), a longitudinal methodology that collects real-time participant data about their thoughts, feelings, or actions, often multiple times a day (Bos et al., 2015; Shiffman & Stone, 1998). This approach, more broadly known as “affective chronometry”, tracks the timing of affective dynamics (Botelho et al., 2018; Davidson, 1998; Dejonckheere et al., 2020; Feidakis, 2011; Heller et al., 2015). For instance, Heller et al. (2015) used EMA to measure the decay of positive emotions after participants received different monetary rewards. Their findings indicated that participants’ emotional reactions approximated an exponential decay process over time, with the initial amplitude and decay rate correlating with brain activity in fMRI scans. This decay pattern is thought to result from how substances move between body areas, such as hormones and neurotransmitters moving through neural and vascular compartments (Ibarra et al., 2020).

We propose that curvilinear decay might be seen in individuals after engaging in pornography use or masturbation. By treating sexual activity as a stimulus that generates affective opponent processes in the body (Henry et al., 2023; Solomon & Corbit, 1974), we hypothesized that an EMA would allow us to observe and quantify both the emotional high during a sexual episode (the “a-process”), and the negative emotional after-effects (the “b-process”) (see Henry et al., 2023 for a more detailed explanation behind this theory). We also hypothesized that these after-effects would follow an exponential decay curve.

### Aims of the Present Study

This study was exploratory in nature, and intended to evaluate the feasibility of quantifying temporal dynamics in the context of pornography use and masturbation in future research. The objectives were:

1. To model the temporal dynamics of pornography users in relation to sexual activities.
2. To determine whether moral incongruence moderated the temporal dynamics produced by pornography use and masturbation.
3. To evaluate the feasibility of conducting an EMA assessment of pornography users, in preparation for a larger cohort of future participants.

## Method

### Participants and Procedure

The study recruited participants through two methods: a pilot study at Auckland University of Technology, New Zealand, using flyers, followed by an exploratory study advertised on the r/pornfree subreddit, known for its anti-pornography stance (r/pornfree, 2022). The pilot study participants received $50 NZD gift vouchers, whereas the exploratory study participants volunteered without financial incentives due to funding limitations.

The study was delivered in two parts: a pre-EMA survey for collecting demographic and descriptive variables, followed by a four-week EMA to monitor temporal dynamics in relation to sexual activity. The pilot study participants also answered a post-EMA survey regarding their EMA experience. Despite different recruitment phases, the core survey questions were consistent between the two studies, allowing us to combine pilot data with the exploratory study data for the final analysis.

Inclusion criteria were proficiency in English; regular internet access and a compatible smartphone; use of pornography at least once in the past month; and minimum age of 18. There was no a priori constraint on sample size. We used Wilcoxon’s rank sum test and Fisher's exact test to compare demographic frequencies between the low and high-incongruence groups; no significant difference in demographic makeup was detected for any metric.

### Measures (Pre-Ecological Momentary Assessment)

The pre-EMA survey was conducted on the online survey and database platform REDCap (Patridge & Bardyn, 2018), containing the measures listed below. A data dictionary with exact item wording for these variables can be found in Appendix B.

#### Demographics and Pre-Screening

Participants were asked generic demographic questions about age, gender, ethnicity, highest level of schooling, employment, relationship status, and religiosity. For screening purposes and to determine baseline sexual activities, participants were asked to state their frequency of pornography use, masturbation, and sexual intercourse over the past month. They were also asked questions regarding the use of any applications or membership in groups aimed at reducing pornography use or masturbation, such as “Reboot” groups.

#### Moral Incongruence and Pornography Consumption

Participants were asked to identify their level of moral incongruence related to masturbation with pornography use, masturbation without pornography use, and pornography use without masturbation. These were measured with one item each (e.g., “I believe that pornography use is morally wrong”) on a scale of 0 (not at all) to 6 (very strongly) based on similar measures in prior studies (Grubbs et al., 2015, 2019a, 2019b; Lewczuk et al., 2020). Typical pornography use levels and PPU screening was performed with the six-item Problematic Pornography Consumption Scale (PPCS-6) (Bőthe et al., 2020, 2021b).

#### Baseline Mental Health Variables

Anxiety and depression disorders were screened with the Hospital Anxiety and Depression Scale (HADS) (Snaith, 2003; Spinhoven et al., 1997). A subset of questions from the Multidimensional Fatigue Inventory (MFI) was used to measure typical levels of mental and motivational fatigue (Smets et al., 1995). Trait levels of guilt and shame proneness were assessed with the eight-item Guilt and Shame Experience Scale (GSES) (Maliňáková et al., 2019). Typical levels of loneliness were assessed with the eight-item short-form UCLA Loneliness Scale (ULS-8) (Hays & DiMatteo, 1987). Participants in a relationship were asked a subset of the seven-item relationship assessment scale to measure relationship connectedness and sexual satisfaction within the relationship (Bőthe et al., 2017; Hendrick et al., 1998). Participants’ social desirability rating, which may indicate biased responses, was assessed with the Brief Social Desirability Scale (BSDS) (Haghighat, 2007).

### Pre-Ecological Momentary Assessment Procedure

EMA data were collected using SEMA3 (Smartphone Ecological Momentary Assessment), an application for longitudinal survey research available on both iOS and Android (O’Brien et al., 2024). Participants engaged in a four-week program, responding to five daily surveys, each lasting 1–2 min with a 3-h response window. Notifications were sent at 9 am, 12 pm, 3 pm, 6 pm, and 9 pm. Variables measured were mood, anxiety, guilt regarding sexual activity, shame regarding sexual activity, loneliness, difficulty thinking, relationship connectedness, and cravings related to pornography use or sexual intercourse. All items were rated on a 0–10 scale, and were specifically developed for this study to capture temporal dynamics while minimizing participant burden. Most items reflected either positive or negative valence; however, mood captured both valences, ranging from 0 (“extremely unhappy”) to 10 (“extremely happy”). A data dictionary with exact item wording for the EMA variables can be found in Appendix C.

Additionally, participants were asked to complete 2–3-min surveys immediately following sexual intercourse, masturbation, or pornography use. They were again asked to rate their cognitive and affective states in the current moment, along with questions about the type, intensity, and duration of their sexual episode, including their highest level of happiness during the episode (using an identical scale to the “mood” variable). They were also asked to categorize their episode as one of the following: sexual intercourse with partner(s), pornography use only, masturbation only, pornography use with masturbation only, pornography use with masturbation and orgasm, or masturbation with orgasm only. Finally, they were asked an open-ended question about the triggers that led to their episode, such as: “What were the events or triggers that led to you using pornography?”, with responses subject to a qualitative content analysis.

No reminder notifications were sent for missed EMA prompts. However, initial notifications remained visible on the participant’s phone screen until they were either dismissed or replaced by the next scheduled EMA prompt. Additionally, upon opening the app, participants were shown a reminder to complete an event-based survey if they had recently engaged in any form of sexual activity. Participants were also informed that their data would be kept confidential within the research team.

### Statistical Analysis

We tested whether cognitive and affective states changed in a predictable way following or preceding pornography use and masturbation. Specifically, we hypothesized that after a sexual episode, these states would show an exponential decay, meaning a sharp change immediately after the event that gradually returned to baseline. For example, we hypothesized that mood would drop sharply in some participants after pornography use before recovering to baseline. We also hypothesized that certain states would show exponential growth leading up to sexual episodes—for example, indicating a buildup of craving or negative emotion leading to the episode.

These temporal dynamics were modelled using the following functions:

- $$w(t) = a{e}^{-bt} + c+\varepsilon $$

for post-episode spike and decay, and.

- $$w(t) = a{e}^{bt} + c+\varepsilon $$

for pre-episode growth, where $w(t)$ is the cognitive or affective state variable; $t$ is time, centered at the sexual episode $(t=0)$; $a$ is the amplitude of the spike in the state variable immediately before or after the sexual event; $b$ is a decay constant governing the rate of change (decay or growth); $c$ is the baseline level of the state variable, and $\varepsilon$ is random noise. When plotted, Eq. 2) is the mirror image of Eq. 1) around the axis at $t=0$.

We compared these models against a null hypothesis model assuming no change over time:

- $$w(t)=d+\varepsilon $$

where $d$ is the average score of the state variable, indicating no effect of the sexual episode on that state.

We used the time stamps of EMA responses to measure temporal dynamics in an approach similar to disease progression modelling (e.g., see Raket, 2020, 2022). This allowed us to model each cognitive and affective state variable as a function of time relative to sexual episodes. By aligning all data points so that each sexual episode occurred at time $t=0$, we constructed a dataset containing aggregated pre- and post-episode observations, centered around the timing of each event. As a result, state ratings fell between two sexual episodes and could be classified as both pre- and post-episode. Therefore, to mitigate the effects of potential cross-contamination, we analyzed results from both the full and truncated datasets, where the truncated dataset excluded any state ratings that occurred closer to a different sexual episode than the one under analysis.

We used Bayesian hierarchical mixed-effects modeling to fit both the exponential and linear models and estimate the parameters $a, b, c$ and $d$. Random intercepts were set for each participant to account for individual differences in temporal dynamics while estimating overall population-level effects. We used the brms package in R v4.3.0 for Bayesian modeling of our data (Bürkner, 2017; R Core Team, 2022). In lieu of traditional p-values, our analysis employed Bayes Factors (BF’s) to quantify the strength of evidence for one model over another (Kruschke & Liddell, 2018). BF’s ranging from 3 to 5 denote “weak” evidence, while 5 to 10 suggests “moderate” evidence, 10–100 represents “strong” evidence, and greater than 100 is”very strong evidence” (Etz & Vandekerckhove, 2016). We reported BF_10_ when evidence favoured the alternate hypothesis that the model was exponential, and BF_01_ when evidence favoured the null hypothesis of no effect. Additionally, we provided 95% Credible Intervals (CI’s) to convey the uncertainty in our estimates (Wagenmakers et al., 2018).

We specified the following bounded priors for parameters: uniform priors for $a$ (− 10 to 10, to cover the entire possible range of initial effect sizes), $b$ (− 10 to 0 for the decay model, and 0 to 10 for the growth model, representing a range of plausible half-lives between approximately five minutes and infinity), and $c$ (0 to 10 to cover the full range of possible state scores). For the null hypothesis (no effect), we used normally distributed priors for $d$ with mean and variance taken from each variable’s empirical distribution. R code used for the modeling is included in the Supplementary Materials.

To test for group differences in temporal dynamics based on moral incongruence, we calculated the difference between each participant's state scores within 12 h of sexual episodes, and the mean of their state scores from timepoints more than 12 h away from any episodes.^2^ This difference gave us an approximate measure of the impact of sexual activity on state scores following episodes, as well as a measure of possible craving effects leading up to episodes. We compared these measures between the low- and high-incongruence groups using Bayesian t-tests (BayesFactor package in R; Morey & Rouder, 2012). For all other group comparisons involving descriptive statistics, we used standard two-tailed independent-samples *t*-tests.

## Results

### Descriptive Statistics and Demographics

Participants’ demographic characteristics are presented in Table 1. The pre-EMA survey was accessed by 91 participants, with 38 (41.8%) completing the entire survey. Of these, 7 (18.4%) were from the pilot study, from whom 5 contributed viable data to the EMA; while 31 (81.6%) participants were from the exploratory study, from whom 17 contributed viable data to the EMA.^3^ Participants responded to survey notifications with a mean of 34.0 min (SD = 35.8). Although SEMA3 rounded response times to the nearest minute, we determined that 92.2% of the 1266 completed EMA surveys were completed within two minutes of being started, and 97.4% within five minutes.

**Table 1:** Demographic data for 22 participants who contributed data to the final ecological momentary assessment

Variable	High moral incongruence, N = 10	Low moral incongruence, N = 12
Age (years)	27.30 (11.19)	29.50 (6.68)
Gender		
Woman	0 (0%)	2 (17%)
Man	10 (100%)	10 (83%)
Gender diverse	0 (0%)	0 (0%)
Prefer not to say	0 (0%)	0 (0%)
Ethnicity		
NZ European	0 (0%)	3 (18%)
Chinese	0 (0%)	1 (6%)
Indian	3 (18%)	0 (0%)
English	2 (12%)	2 (12%)
European/North American Caucasian	7 (41%)	4 (24%)
Middle Eastern	1 (6%)	1 (6%)
Hispanic/Latin American	1 (6%)	3 (18%)
African	0 (0%)	0 (0%)
Other/unspecified	3 (18%)	3 (18%)
Sexual orientation		
Heterosexual	10 (100%)	5 (42%)
Bisexual	0 (0%)	5 (42%)
Homosexual	0 (0%)	1 (8%)
Asexual	0 (0%)	0 (0%)
Unsure	0 (0%)	1 (8%)
Prefer not to say	0 (0%)	0 (0%)
Currently in long term relationship		
Yes—I have been with my current partner for at least 1 year	1 (10%)	3 (25%)
Yes—I have been with my current partner for less than a year	2 (20%)	0 (0%)
No—I do not have a partner currently	7 (70%)	9 (75%)
Prefer not to say	0 (0%)	0 (0%)
Importance of religion in life		
Not at all important	1 (10%)	8 (67%)
Not too important	2 (20%)	3 (25%)
Somewhat important	4 (40%)	1 (8%)
Very important	3 (30%)	0 (0%)
Primary religion		
Catholic (including Roman Catholic and Orthodox)	5 (50%)	2 (17%)
Protestant (e.g. Anglican, Orthodox, Baptist, Lutheran, United Church of Canada)	1 (10%)	1 (8%)
Christian Orthodox	1 (10%)	1 (8%)
Jewish	0 (0%)	0 (0%)
Muslim	0 (0%)	1 (8%)
Sikh	0 (0%)	0 (0%)
Hindu	2 (20%)	0 (0%)
Buddhist	0 (0%)	0 (0%)
Atheist (do not believe in God)	1 (10%)	4 (33%)
Agnostic (believe that existence of God is unknowable)	0 (0%)	3 (25%)
Other	0 (0%)	0 (0%)
Prefer not to say	0 (0%)	0 (0%)
Attendance of religious services in past 12 months		
Never	2 (20%)	9 (75%)
Seldom	0 (0%)	0 (0%)
A few times a year	4 (40%)	3 (25%)
Once or twice a month	1 (10%)	0 (0%)
Once a week	0 (0%)	0 (0%)
More than once a week	3 (30%)	0 (0%)
Frequency of masturbation with pornography use in past 4 weeks		
Never	0 (0%)	2 (17%)
Less than once a week	1 (10%)	0 (0%)
1–2 times a week	3 (30%)	1 (8%)
3–4 times a week	1 (10%)	1 (8%)
5–6 times a week	1 (10%)	4 (33%)
1–2 times a day	3 (30%)	3 (25%)
More than twice a day	1 (10%)	1 (8%)
Frequency of masturbation without pornography use in past 4 weeks		
Never	6 (60%)	4 (33%)
Less than once a week	3 (30%)	4 (33%)
1–2 times a week	0 (0%)	2 (17%)
3–4 times a week	1 (10%)	1 (8%)
5–6 times a week	0 (0%)	0 (0%)
1–2 times a day	0 (0%)	1 (8%)
More than twice a day	0 (0%)	0 (0%)
Frequency of sexual intercourse in past 4 weeks		
Never	8 (80%)	7 (58%)
Less than once a week	1 (10%)	2 (17%)
1–2 times a week	1 (10%)	3 (25%)
3–4 times a week	0 (0%)	0 (0%)
5–6 times a week	0 (0%)	0 (0%)
1–2 times a day	0 (0%)	0 (0%)
More than twice a day	0 (0%)	0 (0%)

Participants were divided by level of moral incongruence with respect to pornography use with masturbation, using a median split. Additional descriptive statistics can be found in Appendix A

Of the participants who contributed EMA data, 20 (90.9%) participants were male, and 16 (72.7%) participants were not in a relationship. Overall compliance rates were 25% for the pilot study, and 29% for the exploratory study. Missing data was not imputed due to its temporal complexity. Given the small sample size, moral incongruence was analyzed categorically using a median split: participants scoring 0–3 on the question about moral incongruence related to masturbation with pornography were classified as low moral incongruence, and those scoring 4–6 as high moral incongruence. Participants in both groups had similarly high PPCS-6 scores, with the high moral incongruence group showing a mean of 30.1 (SD = 5.17) and the low moral incongruence group a mean of 29.3 (SD = 10.71). Median scores were identical (30.5), though the low-incongruence group exhibited greater variability, with scores ranging from 6 to 42 compared to 23 to 38 in high-incongruence participants. Notably, 20 of the 22 participants had a PPCS-6 score of 20 or higher, indicating that the majority of participants in both groups met the criteria for PPU (Bőthe et al., 2020, 2021b).

### Examining Sexual Episodes

**Fig. 1:**
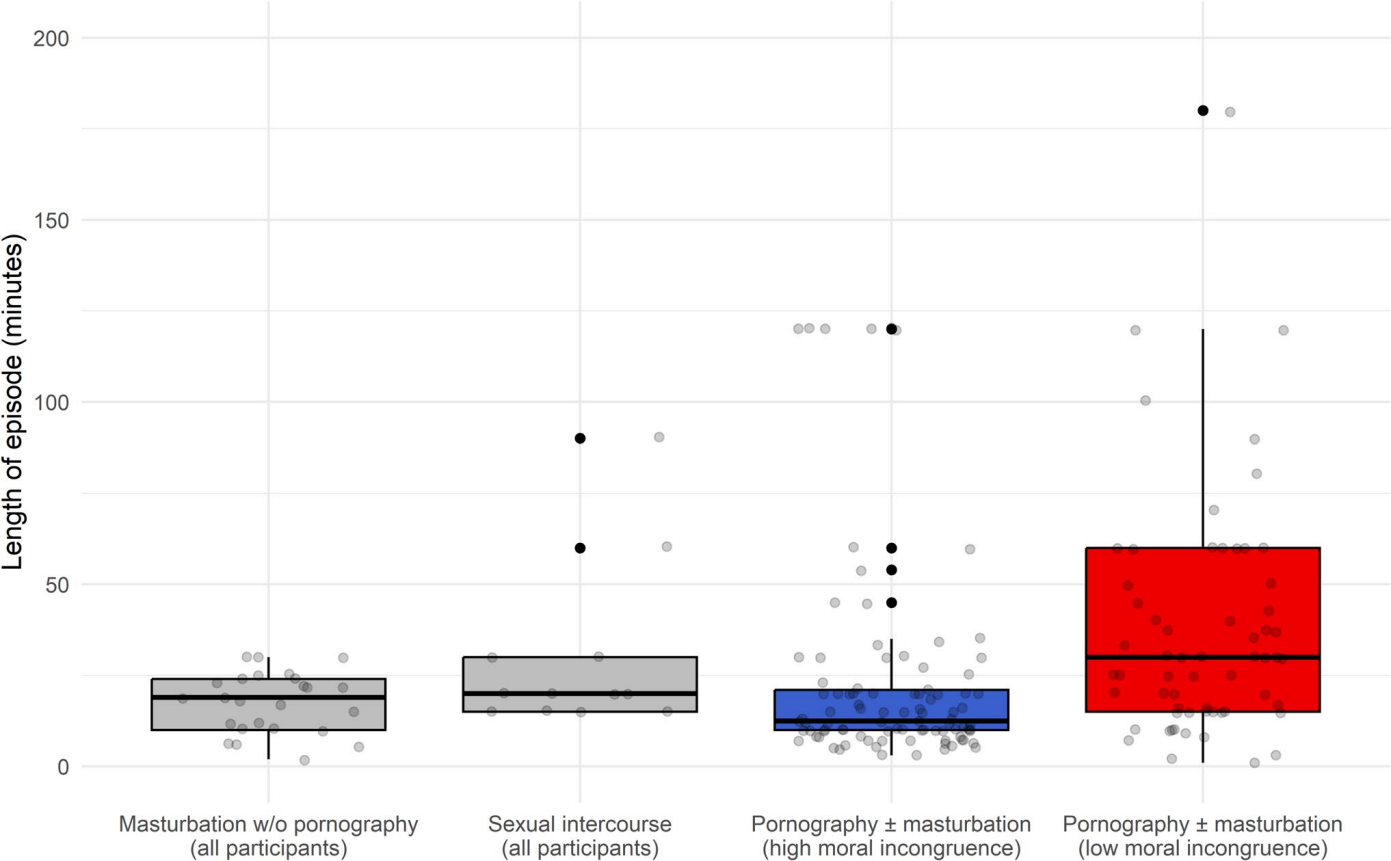
Boxplots with jittered data points showing the length of different types of sexual episode. Due to the small sample size, masturbation without pornography and sexual intercourse could not be categorized into low and high moral incongruence groups. Note that the y-axis has been truncated at 200 min to show differences between groups more clearly. Above this, there was one outlier for the high moral incongruence group (300 min), and four outliers for the low moral incongruence group (240, 480, 480, and 500 min)

In total, there were 162 instances of participants using pornography (with or without masturbation), compared to 26 instances of participants masturbating without pornography, and 13 instances of participants having intercourse with a partner. Figure 1 contains boxplots showing the length of all recorded sexual episodes. In terms of length of episode, masturbation coupled with pornography use had the greatest variation, with a range from 1 to 500 min. The longest episode of masturbation without pornography use was only 30 min long, while the longest episode of sexual intercourse was 90 min long. In contrast, there were 15 episodes of pornography use over 100 min long, and 5 episodes over 200 min long, from more than one participant. This may suggest that some pornography users found content that sustained their interest for considerably longer than masturbation or sexual intercourse alone.

High-incongruence participants had a shorter mean length of pornography use compared to low-incongruence participants (25.93 vs. 61.93 min; *p* = .011, two-tailed *t*-test). Evidence for a significant difference between groups remained even when outliers above 200 min were excluded (22.99 vs. 37.73 min; *p* = .0064, two-tailed *t*-test). Further, there was a moderate negative correlation between moral incongruence and the duration of episodes of pornography use with masturbation (*r* = − .34, *p* < .001). However, no significant correlation was found between moral incongruence score and duration of masturbation-only episodes (*r* = − .21, *p* = .32).

**Fig. 2:**
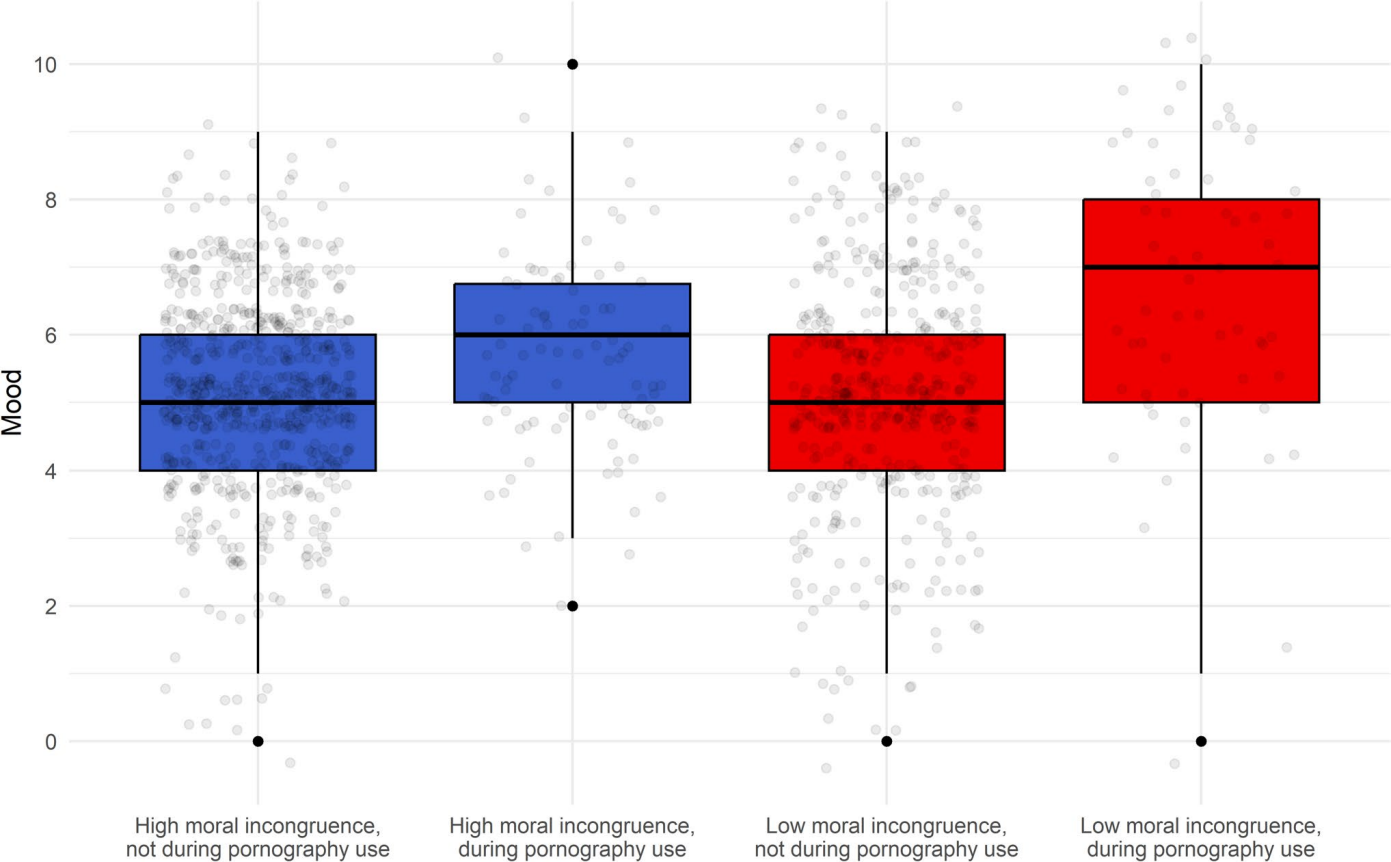
Boxplots with jittered data points comparing the highest mood scores experienced during pornography use (either with or without masturbation or orgasm), and mood scores experienced outside of pornography use, for low and high moral incongruence participants

Figure 2 shows mood scores obtained outside of pornography use, and participants’ highest perceived mood scores during pornography use (either accompanied by or without masturbation or orgasm). Both mood and highest perceived mood were measured on the same 0–10 scale. Wilcoxon rank sum tests with false discovery rate correction applied revealed no significant difference between mood scores obtained outside of pornography use when comparing low- and high-incongruence participants (*p* = 0.44). However, all other comparisons showed significant differences. The most notable difference was observed in the highest mood scores during pornography use between low- and high-incongruence participants (*p* < 0.001). This indicates that participants with low moral incongruence experienced a greater increase in mood during pornography use than high-incongruence participants.

### Descriptive Statistics for EMA Variables

Table 2 contains descriptive statistics for the Pre-Ecological Momentary Assessment variables, filtered by moral incongruence level. Both craving and affect variables exhibited high within-person standard deviations across groups, suggesting dynamic fluctuations in both emotional and motivational states for most participants, consistent with expectations. Craving for pornography and sexual intercourse showed low median values in both groups, suggesting a bursty pattern characterized by intermittent spikes in craving. Guilt, shame, loneliness, and relationship connectedness showed the highest intraclass correlation coefficients (> 0.5 for both groups), suggesting that these variables varied more between individuals than within individuals. In contrast, the remaining variables had intraclass correlation coefficients below 0.5 for both groups, reflecting greater within-person than between-person variability.

**Table 2:** Descriptive statistics for cognitive and affective state variables from ecological momentary assessment

Level of moral incongruence	Variable	M	SD	Median	Within-person SD	Between-person SD	ICC
High moral incongruence	Mood	5.17	1.43	5	1.32	0.60	0.14
	Relationship connectedness	4.39	2.07	4	1.35	1.46	0.53
	Craving for pornography	1.53	2.35	0	2.08	1.52	0.31
	Craving for sex	2.06	2.58	0	1.95	1.75	0.43
	Loneliness	3.98	2.72	4	1.77	1.91	0.53
	Guilt	3.19	2.48	2	1.76	1.93	0.52
	Shame	3.20	2.58	2	1.73	2.22	0.60
	Anxiety	3.01	1.91	3	1.51	1.15	0.36
	Difficulty thinking	2.86	2.18	3	1.83	1.11	0.25
Low moral incongruence	Mood	5.22	1.74	5	1.51	1.06	0.29
	Relationship connectedness	5.31	3.50	5	1.62	2.91	0.76
	Craving for pornography	1.98	2.43	1	2.16	1.63	0.28
	Craving for sex	2.49	2.62	2	2.10	2.04	0.43
	Loneliness	3.39	2.78	3	1.69	2.76	0.71
	Guilt	3.37	2.92	2	1.84	2.74	0.67
	Shame	3.33	3.05	2	1.87	2.66	0.66
	Anxiety	3.30	2.29	3	1.92	1.81	0.41
	Difficulty thinking	3.72	2.45	3	2.30	1.36	0.16

*SD* Standard deviation, *ICC*  Intraclass correlation

**Fig. 3:**
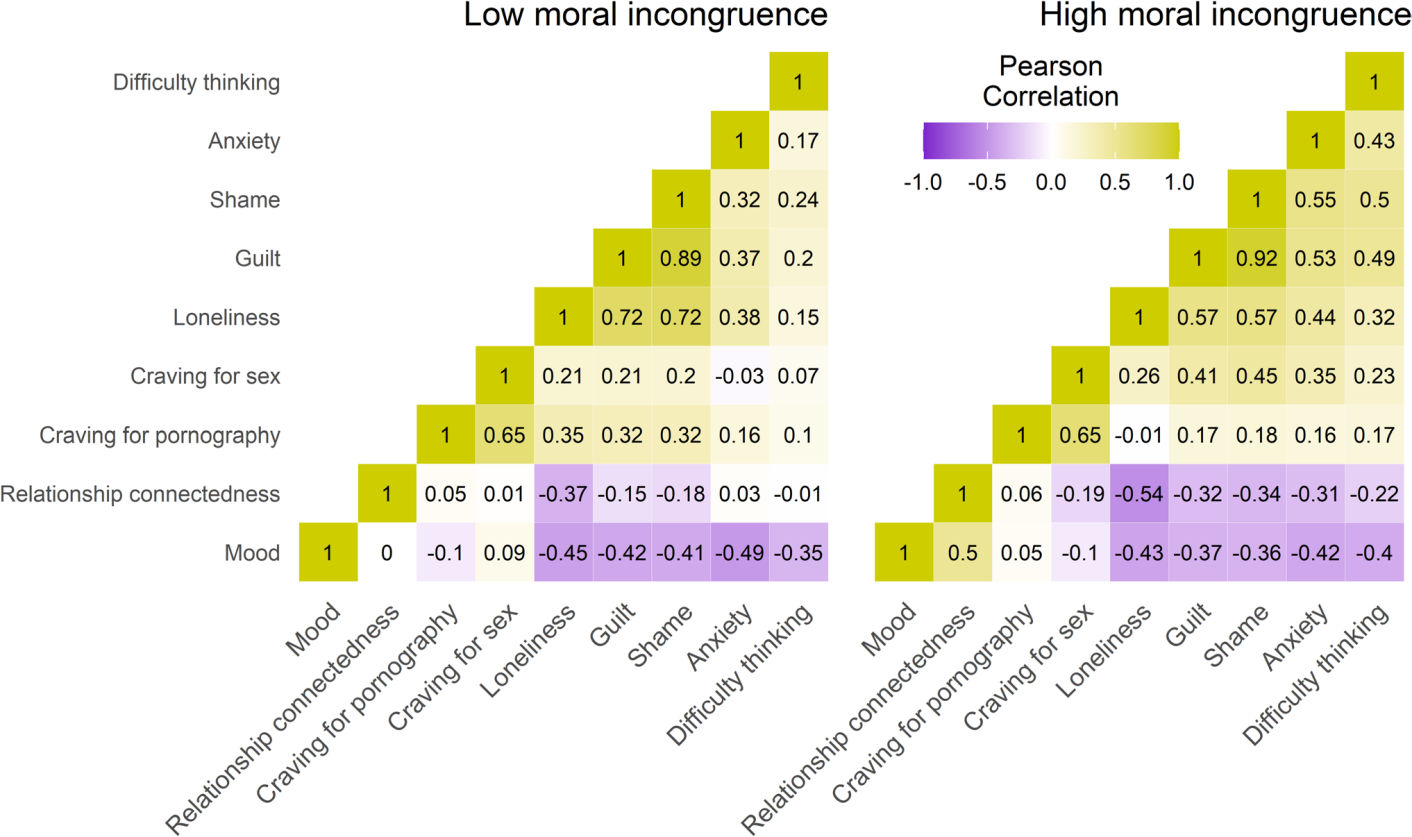
Within-person Pearson correlations between state variables during ecological momentary assessment

Figure 3 shows the within-person Pearson correlations between different cognitive and affective state variables measured during the EMA, using aggregated within-person EMA data. The strongest correlations were observed between guilt & shame, guilt & loneliness, shame & loneliness, and craving for pornography & craving for sexual intercourse. On the other hand, the strongest negative correlations were identified between relationship connectedness & loneliness, mood & loneliness, and mood & anxiety.

### Temporal Dynamics of Cognitive and Affective State Variables

A Table of model coefficients for the Bayesian model fitting process, and plots of selected models, are presented in Appendix A. Our sample size was insufficient to produce a meaningful comparison across different types of sexual episodes; specifically, there was not enough data to differentiate between pornography use combined with masturbation and masturbation alone (Prause, 2017), as only 26 (15%) of the 178 masturbation episodes recorded were not accompanied by pornography use. There were also only 13 episodes of sexual intercourse recorded. Consequently, during the model fitting process, we merged instances of masturbation alone with those involving pornography use, and ignored episodes of sexual intercourse. This allowed us to analyze two groups of sexual episodes:

1. Group A: Episodes involving pornography use, whether or not accompanied by masturbation or orgasm.
2. Group B: All Group A episodes, plus episodes of masturbation without pornography use.

**Fig. 4:**
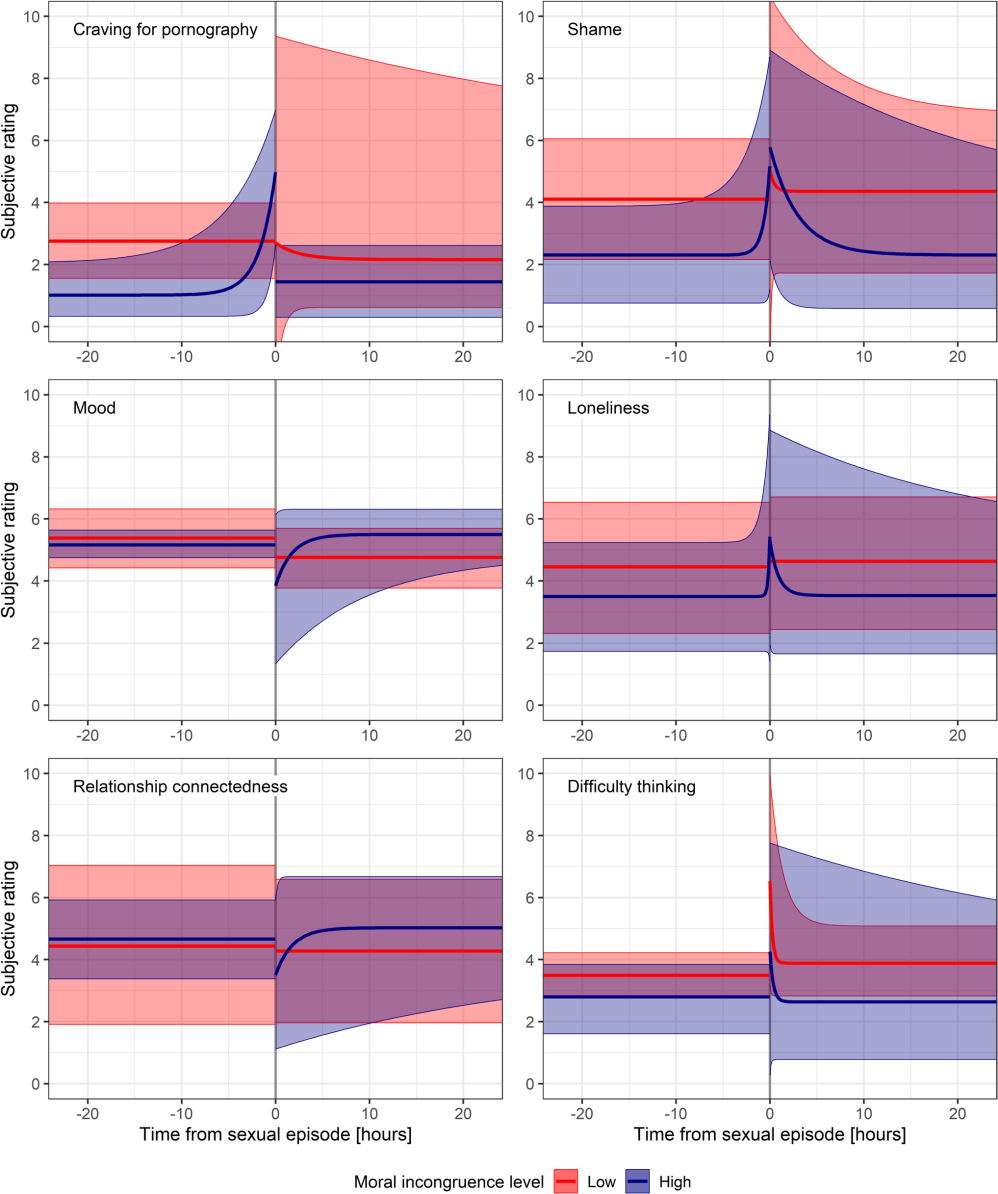
Results from fitting hierarchical exponential models to ecological momentary assessment variables obtained pre- and post-sexual episodes for any episodes of pornography use, either with or without masturbation or orgasm, plus episodes of masturbation without pornography use (i.e., truncated group B data). Only 24 h before and after episodes are shown. Light red graphs are for low moral incongruence participants, while dark blue graphs are for high moral incongruence participants. Error ribbons represent 95% credible intervals. Exponential models are presented in cases where BF_10_ > 1, indicating evidence in favour of the alternative hypothesis (exponential model), while horizontal models indicate cases where BF_01_ > 1, indicating evidence in favour of the null hypothesis (no effect). Opponent process dynamics are apparent in several variables

Figure 4 illustrates the temporal dynamics of key EMA variables both before and after episodes of pornography use, either with or without masturbation or orgasm, plus episodes of masturbation without pornography use (i.e., truncated group B data).

In summary, evidence of opponent process dynamics was strongest for mood, guilt, shame, difficulty thinking, loneliness, and relationship connectedness. This was particularly evident in high-incongruence participants, although some effects were apparent in low-incongruence participants also. Craving sharply increased prior to pornography use and masturbation in high-incongruence participants also. All data, including Bayes factors and model parameters, can be found in Table A2 of Appendix A in the Supplementary Materials, along with associated Figures.

#### Mood

For mood, we found weak to very strong evidence (BF_10_ = 4.19 to 3.20 × 10^6^) that mood decreased following pornography use and masturbation before decaying back to baseline, for high-incongruence participants only. For low-incongruence participants, we found weak to very strong evidence of no effect following sexual episodes. All other models supported no pre- or post-episode effect (BF_01_ ranging from weak to very strong).

#### Difficulty Thinking

We found strong to very strong evidence that difficulty thinking increased for both low- and high-incongruence participants following pornography use and masturbation, before decaying to baseline, particularly for Group B episodes (BF_10_ = 16.32 to 1.12 × 10^5^). Pre-episode effects were generally absent, with inconclusive to strong evidence supporting no increase in difficulty thinking pre-episode for both groups.

#### Relationship Connectedness

We found inconclusive to very strong evidence for a decrease in relationship connectedness that decayed to baseline following sexual episodes for all high-incongruence models (BF_10_ = 2.23 to 5.62 × 10^5^). We also found inconclusive to very strong evidence in favour of a difference in effect sizes between low and high-incongruence participants for these models (BF_10_ = 1.36–3853). However, for all remaining high- and low-incongruence models, we found weak to very strong evidence in favour of no effect prior to or following sexual episodes.

#### Craving for Pornography

We found very strong evidence in favour of a spike in craving for pornography prior to sexual episodes for all high-incongruence models (BF_10_ = 1.17 × 10^8^ to 1.79 × 10^17^). In contrast, for low-incongruence participants, we found inconclusive to very strong evidence in favour of a spike in craving for pornography *after* sexual episodes that decayed to baseline, for all models (BF_10_ = 1.66 to 70.83), indicating possible sensitization. For high-incongruence participants, there was moderate to strong evidence of no effect following sexual episodes (BF_01_ = 6.78 to 84.67), while for low-incongruence participants, evidence favoured no effect prior to sexual episodes (BF_01_ = 1.71 to 5.12).

#### Craving for Sexual Intercourse

Craving for sexual intercourse increased post-episode in high-incongruence participants, with weak to moderate evidence across all but the non-truncated Group A model. Conversely, for low-incongruence participants, evidence ranged from inconclusive to very strong in support of a decrease in craving post-episode, in all but the truncated Group B model. For the remaining models, we found weak to very strong evidence of no pre-episode effect in both groups, except among high-incongruence participants, where there was very strong evidence for an increase in pre-episode craving for intercourse based on non-truncated Group B data (BF_10_ = 3.03 × 10^11^).

#### Shame

We found very strong evidence for all models that shame increased following pornography use and masturbation for high-incongruence participants, before decaying to baseline. For low-incongruence participants, we found moderate to very strong evidence of the same for all but the non-truncated Group B model. Between-group comparisons showed weak to moderate evidence in favour of no difference in effect sizes. However, we also found moderate to very strong evidence for an increase in shame prior to sexual episodes for high-incongruence participants (for all models except truncated Group A data). For low-incongruence participants, we found moderate to very strong evidence in favour of no effect prior to sexual episodes for all models.

#### Guilt

For aggregated within-participant EMA data, guilt was strongly correlated with shame (*r*_low moral incongruence_ = 0.89, *r*_high moral incongruence_ = 0.92). We found very strong evidence for an increase in guilt following pornography use and masturbation for high-incongruence participants, for all but the truncated Group B model. For low-incongruence participants, guilt increased based on non-truncated models, but decreased in the truncated Group B model (BF_10_ = 1997). However, we found moderate evidence in favour of no difference in effect size between low- and high-incongruence participants for all models (BF_01_ = 5.16 to 6.80). We also found very strong evidence for an increase in guilt prior to truncated Group A episodes for high-incongruence participants, but all other models were either inconclusive or showing evidence in favour of no effect for both groups.

#### Loneliness

For loneliness, only the truncated Group B model showed strong evidence of a post-episode increase for high-incongruence participants (BF_10_ = 38.02), along with very strong evidence for a group difference in effect size (BF_10_ = 1.30 × 10^4^). All other models indicated no effect (ranging from inconclusive to very strong evidence) for both groups.

#### Anxiety

Finally, we found inconclusive evidence for an increase in anxiety following Group A episodes for high-incongruence participants, and moderate to very strong evidence in favour of no effect for all remaining models.

All Bayes factors and model parameters can be found in Table A2 of Appendix A in the Supplementary Materials, along with corresponding Figures containing the graphical output of these models.

### Content Analysis of Pornography Use Triggers

Participant’s responses to the open-ended question about triggers leading up to pornography use or masturbation were categorized according to Table 3. We performed an exploratory content analysis of these responses, creating feature categories inductively during the coding process. Although not powered for formal hypothesis testing, these findings offer preliminary insights into some of the more common antecedents of pornography use and masturbation, and may inform the design of more structured, theory-driven trigger assessments in future studies.

**Table 3:** Coding scheme used to categorize open-ended responses about triggers leading up to sexual episodes

Feature category	Type of feature
Sleep	Sleep aid, pre-sleep routine, waking routine, couldn’t sleep, or disturbed sleep
Craving	Craving for pornography, arousal, or ‘horniness’
Bored	Boredom, nothing to do
Stressed	Stress, overwhelm, burnout
Internet/Social Media	Browsing the Internet or social media
Loneliness/Alone	Feelings of loneliness, or being alone/isolated
Tired	Feelings of tiredness
Thoughts	Sexual thoughts or fantasies
Work	Work, school, or studying
Habit	Habit or routine
Bedroom	In bedroom or in bed
Drugs	Alcohol or marijuana
Distraction	Needed a distraction or procrastinating
Interaction	Real-life interaction with attractive person
Anxiety	Feelings of anxiety
Date	Went on a date with no sexual intercourse
Unattractive	Felt unattractive or wanted to feel more attractive
Dream	Sexual dream
Other	Other or unknown triggers

**Fig. 5:**
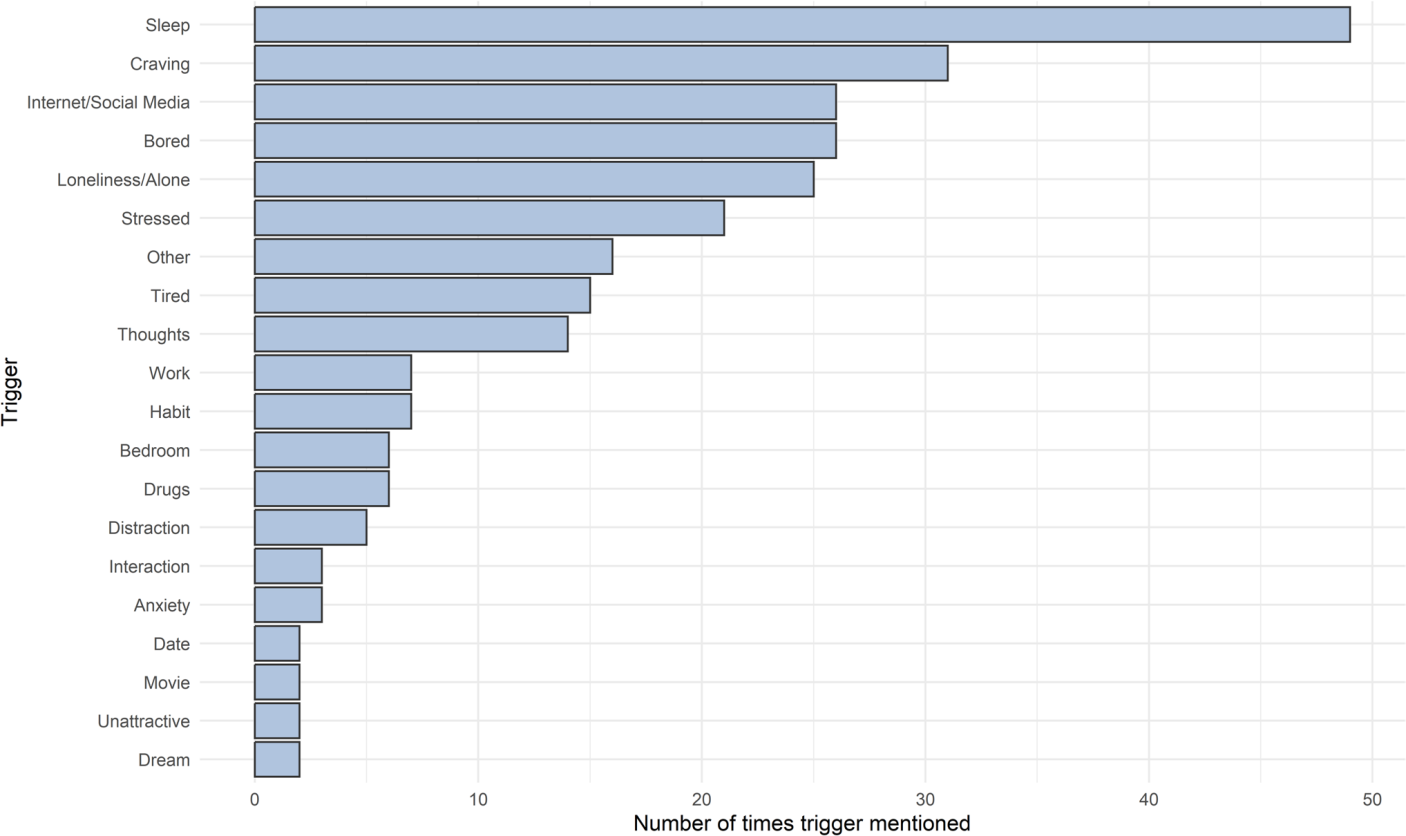
Tally of triggers leading up to pornography use or masturbation. Only triggers with at least two recorded instances were plotted

Data were combined for all sexual episodes and for both high- and low-incongruence participants. The six most mentioned triggers (Fig. 5) were Sleep (49), Craving (31), Internet/Social Media (26), Bored (26), Loneliness/Alone (25), and Stressed (21). Despite Craving, Loneliness, and Anxiety being frequently cited as triggers, they did not show significant increases prior to sexual episodes in any of the quantitative models. Possible reasons for this are discussed in the Section "Clinical and Therapeutic Implications."

## Discussion

To our knowledge, this is the first study to quantify the cognitive and affective dynamics associated with pornography use and masturbation at a high temporal resolution. Overall, participants high in moral incongruence exhibited distinct patterns of affective and cognitive dynamics surrounding pornography use and masturbation compared to those low in moral incongruence. For example, craving increased sharply prior to sexual episodes for high-incongruence participants, while in contrast, craving tended to spike after sexual episodes for low-incongruence participants, suggesting possible sensitization effects. High-incongruence participants also showed consistent elevations in shame, guilt, difficulty thinking, relationship disconnectedness, craving for sexual intercourse, and mood deterioration following pornography use and masturbation, all of which decayed back to baseline over time. Low-incongruence participants, by comparison, experienced smaller or absent changes across most variables. While they did show post-episode increases in shame, guilt, and difficulty thinking, these effects appeared to be weaker or less consistent, with strong evidence for no change in mood or relationship connectedness. Loneliness and anxiety showed limited evidence of change for either group. Together, these results suggest that pornography use and masturbation are more likely to exacerbate—rather than alleviate—negative emotional and cognitive states in high-incongruence individuals. By contrast, low-incongruence individuals appear to experience fewer or more transient disruptions, consistent with a more permissive or less conflicted appraisal of pornography use.

These findings align with and extend prior research indicating that affective and cognitive disturbances associated with pornography use are more pronounced in individuals with high moral incongruence. While previous studies have documented associations between PPU and increased guilt, shame, and distress, most have relied on retrospective or cross-sectional data. By using EMA data, this study was able to trace temporal dynamics in real time, highlighting the transient nature of these affective and cognitive changes. Our observation that low-incongruence participants experienced smaller but measurable increases in shame, guilt, and cognitive disruption also complements findings from Perry (2018), who reported that both low- and high-incongruence individuals may experience pornography-related depression, though via distinct mechanisms: in low-incongruence individuals, depressive symptoms tend to emerge only with high-frequency use. This result, combined with our findings, contribute empirical support to the allostatic opponent process model of pornography use, which posits that frequently repeated rewards may generate escalating b-processes over time, ultimately leading to a shift in hedonic set-point (Henry et al., 2023; Solomon & Corbit, 1974). This would help to explain why low-incongruence individuals tend to experience depression only at high frequencies of pornography use: their b-processes following pornography use may be weak enough to return to baseline after infrequent use, but with repeated high-frequency exposure, these processes may accumulate, resulting in measurable mood disturbances. While this model has been referenced in theoretical discussions of problematic behaviors, they have not yet been operationalized using temporally granular data.

Two unexpected findings emerged from this study. Firstly, both high- and low-incongruence participants reported greater cognitive difficulties following pornography use. This aligns with the “brain fog” phenomenon described in recent qualitative research (Fernandez et al., 2021; Ince et al., 2023), but implies that the phenomenon may arise from neuropsychological mechanisms not tied to moral incongruence. Second, low-incongruence participants showed increased craving for pornography following use–a somewhat paradoxical finding, given that prior research typically frames craving as a precursor to sexual behavior (Allen et al., 2017). This post-episode craving may reflect sensitization processes in low-incongruence participants. Both of these findings warrant further investigation.

### Implications of Temporal Dynamics Analysis

Our findings provide preliminary evidence that moral incongruence moderates the temporal dynamics governing the relationship between pornography use, masturbation, and mental well-being. In particular, this is evidenced by the heightened feelings of guilt and shame experienced by high-incongruence participants after sexual episodes, in contrast to low-incongruence participants. This aligns with Grubbs’ moral incongruence model, which posits that distress linked to pornography use often stems from a conflict between personal behavior and moral beliefs, rather than from compulsive use itself (Grubbs & Perry, 2019; Grubbs et al., 2015). However, the presence of b-process dynamics in low-incongruence participants suggests that additional mechanisms beyond moral conflict may also contribute to negative affective responses.

Recently, Henry et al. (2023) proposed a computational model of opponent processes demonstrating how a frequently repeated behavior may generate b-processes that sum over time, producing hedonic allostasis that can lead to depression. Our findings regarding mood suggest a pattern consistent with this model of allostatic opponent process theory. Specifically, during the sexual episode, high-incongruence participants experienced a peak mood that exceeded baseline (the “a-process”), then experienced a reversal in mood that dropped below baseline and decayed over time (the “b-process”) (Solomon & Corbit, 1974). If this pattern is repeated frequently, one would expect that the b-processes sum over time, leading to a negative shift in one’s hedonic set point. This allostatic pattern may also help to explain how psychological tolerance develops in PPU, whereby increasingly intense or prolonged stimulation may be required to achieve the same peak affective response during sexual activity. Therefore, we believe this is the first paper to demonstrate a possible neuropsychological mechanism by which PPU can contribute to both depression and buildup of tolerance to pornography for people with high levels of moral incongruence. However, further research is needed to validate this theory.

These findings may also help explain Perry's results (2018), which showed that men who morally reject pornography experience depression even with low use, while those who don’t morally reject pornography only experience depression with high-frequency use. This also aligns with Henry et al. (2023) allostatic opponent process theory, which suggests that individuals with low moral incongruence only experience a small b-process after pornography use, although this can lead to hedonic depression due to allostasis at high pornography use frequencies. In contrast, those with high moral incongruence experience a larger b-process, leading to depression even at low pornography use frequencies. These affective and cognitive shifts, though transient in isolation, may compound at high behavioral frequencies, leading to a sustained change in baseline affective and cognitive functioning due to the cumulative buildup of allostatic load.

An emerging symptom linked to pornography use in recent literature is “brain fog” – a subjective state of cognitive impairment involving signs of mental cloudiness, diminished clarity, or difficulty concentrating (Fernandez et al., 2021; Ince et al., 2023). Our data suggest possible evidence of pornography-induced brain fog, based on observed increases in self-reported difficulty thinking following pornography use and masturbation among both low- and high-incongruence participants. For within-person data from high-incongruence participants, difficulty thinking was moderately correlated with shame (*r* = .50), guilt (*r* = .49), decreased mood (*r* = .40), and loneliness (*r* = .32), suggesting the post-episode emotional overwhelm of these symptoms may produce cognitive difficulties akin to brain fog. These preliminary findings, though requiring further investigation, could potentially account for some of the unexplained symptoms (including brain fog) reported by those in online forums such as r/NoFap, a forum dedicated to helping people quit pornography use (Chasioti & Binnie, 2021).

The lack of evidence for a temporal relationship between anxiety and pornography use was surprising, given that several studies have shown a correlation between PPU and increased anxiety (Vieira & Griffiths, 2024). We were also surprised to observe that there was mixed evidence for loneliness increasing prior to pornography use episodes, as there were 25 instances of “Loneliness/Alone” being mentioned as a trigger in the content analysis. The literature suggests a possible bidirectional relationship between loneliness and PPU, where individuals use pornography to cope with loneliness, which may in turn exacerbate feelings of loneliness, especially for those who have difficulty forming relationships (Mestre-Bach & Potenza, 2023; Vescan et al., 2024). One possible explanation is that participants’ post-hoc analysis of the events leading to pornography use differed from the sensations felt prior to the episode. It may be that craving produces a masking effect that makes it difficult to interpret one’s true emotional state in the moment. There may also be superior alternatives to the exponential model for explaining the temporal dynamics of these variables prior to sexual activity.

The results for relationship connectedness support prior studies that indicate that higher pornography use negatively impacts perceived relationship quality, although this effect is moderated by the partner's moral and religious beliefs (Ruffing et al., 2024; Szymanski et al., 2015), and by the partner's awareness of pornography use (Vaillancourt-Morel et al., 2024). Relationship connectedness was inversely correlated with guilt and shame in both groups, but more strongly among high-incongruence participants (guilt: *r* = − 0.32 vs. –0.15; shame: *r* = − 0.34 vs. − 0.18). Only high-incongruence participants showed a negative correlation between relationship connectedness and anxiety (*r* = − 0.31), while no such association was observed in the low-incongruence group (*r* = 0.03). This may suggest that anxiety plays a moderating role in the relationship between moral incongruence and perceived relationship connectedness. In high-incongruence individuals, long-term pornography use may evoke heightened baseline anxiety, in turn reducing feelings of relationship connectedness. For low-incongruence individuals, the absence of anxiety in this context may help to preserve relationship quality, despite feelings of guilt or shame. However, further research is needed to confirm these findings.

The evidence for an increase in craving for pornography or sexual intercourse prior to sexual episodes for high-incongruence participants made sense, given that craving incubation prior to addictive behaviors such as pornography use is well documented (Allen et al., 2017; Fernandez et al., 2021; Kober et al., 2010; Potenza, 2008; Starcke et al., 2018; Venniro et al., 2021; Volkow et al., 2011). However, we were surprised to find that the same spike in craving was not observed prior to sexual episodes for low-incongruence participants. It may be that craving is exacerbated by overall feelings of lowered mood and relationship connectedness, which was not detected following sexual episodes in low-incongruence participants. However, given that these variables did not decrease just prior to sexual episodes, this interpretation remains uncertain. Furthermore, for some models, we detected increases in craving for pornography after sexual episodes for low-incongruence participants, and increases in craving for sexual intercourse after sexual episodes for high-incongruence participants, which was unexpected. Further research is needed to replicate these paradoxical findings and, if confirmed, to identify their underlying mechanisms.

#### Length and Enjoyment of Sexual Episodes

The difference in length and enjoyment of pornography use episodes between low- and high-incongruence participants may suggest different motivations for use. One hypothesis could be that high-incongruence participants were more likely to use pornography quickly to alleviate unpleasant feelings (such as low mood), while low-incongruence participants might use pornography to alleviate boredom or for extended bouts of pleasure. Maitland and Neilson (2023) showed that individuals who use pornography mainly for enjoyment have social well-being levels similar to those who do not use pornography, while individuals with varied motivations for pornography use and associated negative outcomes often struggle with social well-being, which affects their intimate relationships. Hence, motivation for use may play a role in determining whether one’s pornography use is problematic.

However, our results also possibly show a benefit to moral incongruence in terms of unproductive time saved, since there was a pronounced difference in mean length of pornography use episodes when comparing low to high moral incongruence individuals (61.01 vs. 25.93 min). These results may also support the “supernormal stimulus” hypothesis, which suggests that pornography hijacks the human’s sexual drive with its endless novelty capable of maintaining sexual arousal for unusually long periods (Barrett, 2010; Hilton, 2013; Koukounas & Over, 2000). This could account for the unusually long pornography use sessions observed in our data, with 15 episodes lasting over 100 min and 5 exceeding 200 min—reported by more than one participant.

### Implications of Qualitative Analysis

To not overburden participants, we prioritized certain variables in the EMA analysis and excluded others. However, the content analysis identified several of these excluded variables, such as stress, tiredness, boredom, intrusive sexual thoughts, Internet and social media use, and timing factors (e.g., before sleep, just after waking up, or after work). Future studies could ask about these factors either before or during the EMA, to integrate them into the models. In particular, seasonal variables such as participants’ Internet use, work and sleep schedules should be examined. Furthermore, variables that were specific to participants in a relationship were excluded in favour of the more generic “relationship connectedness” variable, which could be applied to both partnered and unpartnered individuals. Future research should examine how relationship-specific variables such as “relationship satisfaction” change following sexual activity also.

Several participants reported that unintentionally viewing sexual content on the Internet or social media was a trigger for them, with YouTube, Reddit, Instagram, and Twitter being specific platforms mentioned. In the study by Fernandez et al. (2021), electronic media (e.g., “Dating sites, Instagram, Facebook, movies/TV, YouTube, online ads”) were the most common sources of external triggers leading to relapse for members of Reboot Nation. This suggests a growing problem for those struggling with PPU, as casual Internet browsing may pose a high risk of triggering pornography use. In our study, 27% of the EMA participants reported using an app or software to block sexual content on their devices. This aligns with a content analysis by Henry et al. (2022), which found that content-blocking apps were the most commonly downloaded to reduce PPU. More research is needed to study the effectiveness of these apps, as they may play a key role in shielding individuals from unwanted exposure to sexual material.

Future research should consider adding both proximal and distal qualitative markers (Witkiewitz & Marlatt, 2007), such as post-episode questions about how participants feel immediately after pornography use. This could help clarify the sensations experienced and explain possible links between well-studied variables, such as guilt and shame, and less well-examined variables, such as “brain fog”. Additionally, studying the content of the pornography used is important, as different types have been linked to varying levels of sexual satisfaction (Nolin et al., 2025) and may affect mental health differently. However, since EMA is more intensive than other survey methods, researchers must be careful not to burden participants with too many questions. For this reason, and due to predicted statistical limitations and low sample size, we selected EMA variables that were less likely to show seasonal patterns. Future research should consider including these variables and controlling for seasonal effects. For example, although we excluded “boredom” due to its likely association with work hours, the qualitative analysis showed its importance as a trigger for specific participants. Masturbation as a stress-relief mechanism is also well-documented across genders (Csako et al., 2022; Hevesi et al., 2023; Wehrli et al., 2024). Moreover, our findings highlighted the role of sleep and work schedules in triggering cravings, with pornography use often occurring as a sleep aid or after waking up, and for stress relief post-work.

### Clinical and Therapeutic Implications

If validated, these findings have several key implications for clinical practice. Firstly, this study introduces a modeling framework that, for the first time, may allow clinicians to quantify how moral incongruence and pornography use frequency interact to produce negative mental health outcomes—not just over the long term, but dynamically, as a function of the time that has passed since one’s most recent sexual episodes. For example, such a model could allow clinicians to estimate the intensity of depressive symptoms a patient is likely to experience, based on their level of moral incongruence and typical frequency of pornography use. This technique also provides a more coherent educational framework for explaining how high-frequency pornography use may become harmful for certain individuals, particularly those with strong moral or religious beliefs.

The identification of opponent process dynamics in several state variables also highlights opportunities for anticipatory interventions. Clinicians could use the opponent process model of pornography use to predict the best timing for emotion regulation strategies, such as cognitive reframing, self-compassion exercises, or behavioral activation, immediately after pornographic episodes. Likewise, this model suggests that a self-applied EMA could be used as a tool for pre-emptive craving management. The high temporal resolution of the data allows for improved identification of trigger patterns, which could inform more personalized relapse prevention plans; such data could even be integrated into a mobile application in the form of a self-regulated just-in-time adaptive intervention (Nahum-Shani et al., 2018), where the user records their moment-to-moment craving levels in the app, and in turn receives context-sensitive notifications to change their environment, to exercise, or to practice mindfulness or urge-surfing to interrupt escalating cravings. This approach may be particularly valuable for individuals who are afraid to discuss their pornography use with a therapist, instead preferring self-help interventions.

Finally, transdiagnostic versions of the opponent process model may be applicable to other forms of digital overuse, such as social media or smartphone engagement, binge eating, or gambling, which may involve similar affective trajectories to pornography use. For example, recent evidence suggests that moral incongruence plays a part in gambling addiction also (Grubbs et al., 2022a, 2022b), raising the question of whether PPU and gambling disorders share some common mechanisms. Thus, validating this model for PPU provides a framework for comparative analyses of temporal dynamics across many problematic behaviors.

### Limitations

We must be cautious about claiming direct causal effects due to the observational nature of this research. While pornography use in some participants precedes negative emotional states, there may be mediating factors involved. For instance, in high moral incongruence participants, pornography use might initially increase guilt and shame, which then lead to increased loneliness, difficulty thinking, and craving for sexual intercourse, along with worsened mood and relationship connectedness. Furthermore, the differences in temporal dynamics between high and low moral incongruence groups could be influenced by confounding factors. For example, 100% of the high moral incongruence group identified as heterosexual, whereas the low moral incongruence group was more diverse, with 58.3% of this group identifying as bisexual, homosexual, or unsure of their sexual orientation. This variation might be related to religious beliefs, as high moral incongruence participants were generally more religious. Additionally, levels of sexual functioning and sexual distress were not assessed, which limits our ability to contextualize participants’ cognitive and emotional responses in terms of underlying sexual satisfaction or dysfunction. Thus, the observed differences may partly reflect variation in sexual orientation, religiosity, or unmeasured factors such as sexual functioning or distress, rather than solely moral incongruence (Fisher et al., 2019). Future studies should explicitly assess these variables to clarify their role in modifying temporal dynamics.

Participants were subject to self-selection and self-report biases, having mainly come from the r/pornfree subreddit, which is generally anti-pornography but is less critical of masturbation (r/pornfree, 2022). Although we aimed to include diverse moral incongruence values, the relationship between moral incongruence and study participation is unclear. For example, potential participants with faith practices might find questions about sexual intercourse inappropriate or objectifying, which could affect their willingness to share experiences, especially if they feel shame. The Hawthorne effect—where individuals alter their behavior because they know they are being observed—may also have influenced participant engagement and responses (Schofield et al., 2019). For instance, participants with high moral incongruence may have been less likely to report a particularly shameful sexual episode in the EMA. Additionally, 20 out of 22 participants who contributed EMA data had a moral incongruence score of 20 or higher on the PPCS-6, exceeding the threshold for PPU (Bőthe et al., 2020, 2021b). This highlights the need for additional data from high- and low-incongruence individuals who do not meet the criteria for PPU, in order to isolate the effects of moral incongruence from those of problematic use.

We encountered high participant attrition rates and low compliance rates during the EMA, which is consistent with previous EMA studies (Bőthe et al., 2021). It is hard to say how financial incentives affected participation; in fact, the exploratory study with no financial incentive had a higher rate of compliance (29%) compared to the pilot study which had a financial incentive (25%), although these figures include participants who did not complete any EMA surveys. Also, participants were drawn from different populations for these two studies, making them difficult to compare.

Our study was limited by sample size, increasing the uncertainty of parameter estimates in our hierarchical modelling. We were also unable to obtain enough data to analyse the effects of masturbation on its own versus masturbation and pornography use combined (Prause, 2017). There were only 26 instances of masturbation without pornography use, making it impossible to compare this against pornography use with masturbation (152 instances), or even against pornography use by itself (10 instances) or sexual intercourse (13 instances). Future research should account for these low rates and factor them into power calculations to enable suitably powered comparisons between these categories.

Efforts to incorporate participant-level random effects led to overfitting and convergence challenges in certain exponential models, likely stemming from insufficient data. In these cases, the Bayes Factor leaned heavily in favour of the null hypothesis of no effect. It is also possible that the temporal resolution of the EMA was too coarse to detect rapid fluctuations in temporal dynamics. However, by aggregating pre- and post-episode affective scores across multiple sexual episodes, the timing of episodes was effectively randomised relative to EMA prompt times. This design increased the effective sampling frequency across participants and episodes, partly mitigating the limitations in capturing high-frequency changes.

Furthermore, the decay periods for EMA variables following sexual episodes appeared unusually short in some models. In preliminary analyses, we generated models without accounting for individual effects and found that the decay period often spanned several hours to days. This discrepancy may indicate a bias in the models towards certain participants’ data due to the small sample size (McNeish & Stapleton, 2016). To address these issues, future EMA studies should aim for larger sample sizes and include participants as random effects to enhance model convergence and parameter estimation.

### Conclusion

In conclusion, this exploratory study demonstrates the value of using high-frequency EMA data to quantify the impact of moral incongruence on the temporal dynamics that accompany pornography use and masturbation. Compared to low-incongruence participants, those with high moral incongruence experienced more intense and frequent spikes in shame, guilt, difficulty thinking, relationship disconnectedness, craving for sexual intercourse, and mood deterioration immediately after pornography use and masturbation. These findings are consistent with opponent process theory, suggesting a novel mechanism linking problematic pornography use to depression. However, our small, self-selected sample and reliance on self-report measures may limit the generalizability of these findings and introduce reporting biases. Future research should attempt to replicate these findings in larger, more diverse cohorts—including individuals with more normative pornography use—to determine whether opponent process dynamics generalize to pornography use and masturbation more broadly.

## Supplementary Information

Below is the link to the electronic supplementary material.

Supplementary file1 (PDF 3982 KB)

Supplementary file2 (PDF 75 KB)

Supplementary file3 (PDF 152 KB)

## Data Availability

The code that supports the findings of this study is available in the Open Science Framework (OSF) website at https://osf.io/u7xfq/, https://doi.org/10.17605/OSF.IO/U7XFQ.

## Footnotes

1 Throughout this paper, we distinguish between general pornography use (which may be normative or non-problematic) and problematic pornography use (PPU), which refers to pornography consumption that causes distress or functional impairment.

2 This threshold was chosen because, in most cases, the exponential models decayed to near baseline within 12 h.

3 Data from a sixth contributing participant were excluded due to a software error in the SEMA3 app that compromised the reliability of their data.
