# Supplementary material for: Quantifying Opponent Process Dynamics in Pornography Use and Masturbation: An Exploratory Ecological Momentary Assessment Study
Source: Arch Sex Behav. 2025 Nov 21;54(9):3313–34. doi: 10.1007/s10508-025-03287-z (PMC12675693; doi:10.1007/s10508-025-03287-z)
Supplement: Supplementary file 1 — Supplementary file1 (PDF 3982 KB) [file 10508_2025_3287_MOESM1_ESM.pdf]

Appendix A

1.1 Additional descriptive statistics

Table A1 contains additional descriptive statistics that were not included in the body of the main article.

We detected a very strong correlation between responses to the question 'How important is religion in your life?' and higher moral incongruence scores for masturbation with pornography use ($r = 0.70$, $p = 0.0032$), as well as strong correlations with moral incongruence scores for pornography use by itself ($r = 0.60$, $p = 0.0029$) and with moral incongruence scores for masturbation by itself ($r = 0.67$, $p < 0.001$).

We detected a moderate correlation between participants' PPCS-6 scores (measuring PPU tendencies) and their ULS-8 loneliness scores ($r = 0.47$, $p = 0.027$) and HADS depression scores ($r = 0.47$, $p = 0.028$). We did not find evidence for a correlation between PPCS-6 scores and MFI mental fatigue scores ($r = 0.43$, $p = 0.051$), HADS anxiety scores ($r = 0.30$, $p = 0.18$), GSES shame scores ($r = 0.42$, $p = 0.053$) or GSES guilt scores ($r = 0.36$, $p = 0.10$).

For participants with high moral incongruence, the mean score for moral incongruence regarding pornography use combined with masturbation (5.60) was significantly higher than the mean score for moral incongruence regarding masturbation alone (3.40, $p = 0.0098$). For low moral incongruence participants, the difference was not significant (1.00 vs 0.33, $p = 0.14$).

Table A1: Additional demographic data for 22 participants who contributed data to EMA, divided by level of moral incongruence with respect to pornography use with masturbation.

Variable	High moral incongruence, N = 10	Low moral incongruence, N = 12
**Currently active member of any online or in-person groups to reduce pornography use or masturbation frequency**	6 (60%)	8 (67%)
**Total relationship satisfaction score (0-8)**	3.67 (0.58)	5.00 (1.00)
**Marital status**		
Married/civil union/de facto	1 (10%)	1 (8.3%)
Separated/divorced/widowed	1 (10%)	0 (0%)
Never married (single)	8 (80%)	11 (92%)
Prefer not to say	0 (0%)	0 (0%)
**Highest level of education**		
Primary school/Elementary school	0 (0%)	0 (0%)
Intermediate school/Middle school	0 (0%)	0 (0%)
High school	0 (0%)	2 (17%)
Trade or technical qualification	0 (0%)	0 (0%)
Diploma or certificate	0 (0%)	0 (0%)
University/college (undergraduate)	8 (80%)	8 (67%)
University/college (postgraduate)	2 (20%)	2 (17%)
**Employment status**		

Variable	**High moral incongruence**, N = 10	**Low moral incongruence**, N = 12
Full-time: Paid employment for >=30 hrs a week	4 (40%)	6 (50%)
Part-time: Paid employment for 1 to less than 30 hrs a week	1 (10%)	3 (25%)
Not in paid employment or paid employment for less than 1 hour per week	5 (50%)	3 (25%)
Retired	0 (0%)	0 (0%)
**Online groups/forums used (can select more than one)**		
r/nofap, r/nofapteens or r/nofapchristians on reddit.com or nofap.com	1 (10%)	4 (27%)
r/pornfree or r/pornfreewomen on reddit.com	6 (60%)	8 (53%)
Fight The New Drug (FTND) - fightthenewdrug.org	3 (30%)	0 (0%)
Your Brain On Porn (YBOP) - yourbrainonporn.com	0 (0%)	1 (7%)
Sexaholics Anonymous	0 (0%)	0 (0%)
Other	0 (0%)	2 (13%)

Variable	High moral incongruence, N = 10	Low moral incongruence, N = 12
**Currently using an app/software to help reduce pornography use or masturbation frequency**	6 (60%)	4 (36%)
**Reason for using app/software (can select more than one)**		
To block sexual content on the internet or my device	4 (31%)	2 (25%)
To access tutorials/coaching sessions to help me reduce pornography use/masturbation	2 (15%)	0 (0%)
To keep track of my 'streak length' (i.e. how long since I last used pornography/masturbated	4 (31%)	2 (25%)
To connect with an 'accountability buddy' who checks my progress reducing pornography use/masturbation	1 (8%)	1 (12%)
To keep track of my mental health over time (e.g. tracking mood, anxiety or stress levels on a daily basis	0 (0%)	0 (0%)
To keep track of my financial savings from not using pornography	0 (0%)	1 (12%)

Variable	High moral incongruence, N = 10	Low moral incongruence, N = 12
To distract me from using pornography or masturbating (e.g. through playing games, meditating, or using a 'panic button'	2 (15%)	1 (12%)
Other	0 (0%)	1 (12%)
**Total HADS depression score (0-21)**	6.30 (5.14)	8.08 (4.48)
**Total HADS anxiety score (0-21)**	9.00 (3.83)	9.58 (4.19)
**Total MFI score (4-20)**	13.10 (2.64)	13.55 (4.16)
**Total BSDS score (0-4)**	1.67 (1.41)	1.67 (0.89)
**Total GSES score (8-32)**	23.20 (3.79)	22.08 (5.74)
**Total ULS-8 score (8-32)**	22.70 (6.68)	21.17 (7.54)
**Total PPCS-6 score (6-42)**	30.10 (5.17)	29.33 (10.71)
**Moral incongruence score for pornography use without masturbation**		
0 - Not at all	0 (0%)	7 (58%)
1	0 (0%)	2 (17%)
2	0 (0%)	0 (0%)
3 - Somewhat	0 (0%)	2 (17%)
4	2 (20%)	1 (8.3%)

Variable	High moral incongruence, N = 10	Low moral incongruence, N = 12
5	3 (30%)	0 (0%)
6 - Very strongly	5 (50%)	0 (0%)
**Moral incongruence score for masturbation without pornography use**		
0 - Not at all	1 (10%)	9 (75%)
1	1 (10%)	2 (17%)
2	1 (10%)	1 (8.3%)
3 - Somewhat	3 (30%)	0 (0%)
4	1 (10%)	0 (0%)
5	0 (0%)	0 (0%)
6 - Very strongly	3 (30%)	0 (0%)
**Moral incongruence score for masturbation with pornography use**		
0 - Not at all	0 (0%)	7 (58%)
1	0 (0%)	1 (8.3%)
2	0 (0%)	1 (8.3%)
3 - Somewhat	0 (0%)	3 (25%)
4	1 (10%)	0 (0%)

**Variable**	**High moral incongruence**, N = 10	**Low moral incongruence**, N = 12
5	2 (20%)	0 (0%)
6 - Very strongly	7 (70%)	0 (0%)

1.2 Model parameters produced by Bayesian analysis

Table A2 contains all model parameters produced from attempting to fit hierarchical exponential models to mental health scores obtained pre- and post-sexual episode via Bayesian modeling.

Group A: any episodes of pornography use, either with or without masturbation or orgasm. Group B: any episodes of pornography use, either with or without masturbation or orgasm, plus episodes of masturbation without pornography use.

Table A2: Model parameters produced from Bayesian modeling process. For the final column, Bayesian t-tests were only performed if the Bayes Factor in favour of the exponential model was greater than 1 for either of the low or high moral incongruence levels.

State variable	Pre- or post-episode	Type of sexual episode	Truncation	Moral incongruence level	a, amplitude of exponential model [95% CI]	b, decay constant of exponential model [95% CI]	c, offset of exponential model [95% CI]	d, intercept of linear model [95% CI]	Posterior probability for exponential model	Posterior probability for linear model	Bayes Factor in favour of exponential model	Bayes Factor in favour of difference in effect sizes between high and low moral incongruence participants
Anxiety	*Post-episode*	*Group A*	*No*	*High moral incongruence*	0.3258 [-1.395, 2.67]	-6.264 [-9.826, -1.358]	3.202 [2.161, 4.156]	3.199 [2.291, 4.078]	0.6445	0.3555	1.744	0.4346
				Low moral incongruence	-0.7463 [-3.082, 1.361]	-6.609 [-9.87, -1.927]	3.8 [2.849, 4.795]	3.721 [2.744, 4.699]	0.04838	0.9516	0.05016	
			Yes	*High moral incongruence*	0.3703 [-1.522, 2.759]	-4.133 [-9.719, -0.05776]	3.036 [1.671, 4.294]	3.166 [2.029, 4.303]	0.564	0.436	1.284	4.161
				Low moral incongruence	-0.4138 [-2.72, 1.807]	-5.453 [-9.831, -0.02377]	3.543 [1.88, 5.081]	3.4 [1.964, 4.705]	0.04887	0.9511	0.05257	
		Group B	*No*	*High moral incongruence*	-0.3899 [-1.606, 1.502]	-5.615 [-9.811, -1.407]	4.116 [2.398, 6.916]	3.122 [2.27, 3.993]	0.007504	0.9925	0.004168	
				Low moral incongruence	-0.5904 [-2.404, 1.108]	-6.267 [-9.828, -0.017]	3.874 [2.884, 4.964]	3.749 [2.803, 4.686]	0.0391	0.9609	0.0394	
			Yes	*High moral incongruence*	-0.05286 [-1.366, 1.763]	-5.042 [-9.743, -0.2978]	4.484 [2.095, 8.325]	3.146 [2.074, 4.306]	5.416e-05	0.9999	7.526e-05	
				Low moral incongruence	-1.042 [-3.219, 1.413]	-4.971 [-9.79, -1.19]	4.698 [2.217, 8.204]	3.389 [2.034, 4.678]	5.535e-05	0.9999	0.0001142	
	Pre-episode	*Group A*	*No*	*High moral incongruence*	-0.9165 [-2.974, 1.419]	6.968 [2.45, 9.873]	3.146 [2.177, 4.182]	3.07 [2.073, 3.985]	0.06915	0.9308	0.07743	
				Low moral incongruence	-0.7996 [-4.654, 4.172]	6.359 [1.741, 9.833]	2.941 [1.504, 4.533]	3.397 [2.11, 4.587]	0.003926	0.9961	0.004399	
			Yes	*High moral incongruence*	-0.8229 [-3.157, 1.199]	6.692 [2.419, 9.836]	4.183 [2.326, 7.08]	3.181 [2.201, 4.19]	4.16e-08	1	5.345e-09	
				Low moral incongruence	-0.1887 [-4.666, 4.702]	6.114 [0.9497, 9.837]	3.424 [2.189, 4.507]	3.377 [2.088, 4.462]	0.2337	0.7663	0.3239	
		Group B	*No*	*High moral incongruence*	-0.8759 [-2.991, 1.522]	6.96 [2.442, 9.879]	3.123 [2.118, 4.145]	3.101 [2.148, 4.016]	0.07623	0.9238	0.07782	

**State variable**	**Pre- or post-episode**	**Type of sexual episode**	**Truncation**	**Moral incongruence level**	**a, amplitude of exponential model [95% CI]**	**b, decay constant of exponential model [95% CI]**	**c, offset of exponential model [95% CI]**	**d, intercept of linear model [95% CI]**	**Posterior probability for exponential model**	**Posterior probability for linear model**	**Bayes Factor in favour of exponential model**	**Bayes Factor in favour of difference in effect sizes between high and low moral incongruence participants**
				Low moral incongruence	-1.632 [-4.595, 2.239]	6.463 [1.532, 9.853]	3.682 [2.473, 4.89]	3.706 [2.438, 5.072]	0.1631	0.8369	0.2056	
			Yes	*High moral incongruence*	-1.222 [-3.492, 1.305]	6.874 [2.143, 9.841]	3.2 [2.044, 4.265]	3.17 [2.184, 4.167]	0.1687	0.8313	0.2073	
				Low moral incongruence	-1.143 [-4.588, 2.224]	5.446 [2.163, 9.811]	4.846 [2.443, 8.791]	3.516 [2.4, 4.689]	0.007032	0.993	0.01026	
Craving for pornography	*Post-episode*	*Group A*	*No*	*High moral incongruence*	0.9145 [-1.741, 3.679]	-6.382 [-9.773, -0.6806]	2.472 [0.9044, 4.247]	1.88 [0.8747, 2.993]	0.00626	0.9937	0.01181	0.1485
				Low moral incongruence	0.8659 [-2.696, 4.306]	-0.8743 [-6.917, -0.0795]	2.418 [1.482, 3.513]	2.426 [1.353, 3.544]	0.6887	0.3113	1.661	
			Yes	*High moral incongruence*	0.1617 [-1.423, 2.061]	-5.943 [-9.879, -0.9108]	1.508 [0.4221, 2.672]	1.494 [0.441, 2.656]	0.02034	0.9797	0.02069	0.4670
				Low moral incongruence	0.4333 [-3.168, 3.973]	-1.682 [-7.836, -0.09355]	1.948 [0.6448, 3.256]	2.056 [0.9049, 3.251]	0.6198	0.3802	2.021	
		Group B	*No*	*High moral incongruence*	-0.1445 [-1.713, 2.175]	-5.602 [-9.797, -0.2871]	2.008 [0.9972, 3.106]	1.852 [0.8585, 2.849]	0.1267	0.8733	0.1475	0.2200
				Low moral incongruence	1.103 [-2.105, 3.973]	-0.5879 [-6.069, -0.06977]	2.393 [1.515, 3.32]	2.591 [1.415, 3.78]	0.988	0.01201	70.83	
			Yes	*High moral incongruence*	0.07026 [-1.474, 1.964]	-6.051 [-9.83, -1.214]	1.459 [0.3214, 2.654]	1.444 [0.297, 2.615]	0.02115	0.9788	0.0194	452.1
				Low moral incongruence	0.5355 [-2.789, 3.911]	-0.4033 [-1.523, -0.02186]	2.163 [0.6181, 5.452]	2.062 [0.9872, 3.199]	0.9573	0.04266	21.28	
	Pre-episode	*Group A*	*No*	*High moral incongruence*	3.828 [2.052, 4.921]	0.4949 [0.2276, 0.9649]	1.587 [0.5713, 2.748]	1.934 [0.9526, 3.028]	1	4.736e-18	1.791e+17	0.1863
				Low moral incongruence	1.792 [-3.842, 4.785]	3.688 [0.05602, 9.73]	1.994 [0.7922, 3.141]	2.148 [1.046, 3.211]	0.3901	0.6099	0.5373	

State variable	Pre- or post-episode	Type of sexual episode	Truncation	Moral incongruence level	a, amplitude of exponential model [95% CI]	b, decay constant of exponential model [95% CI]	c, offset of exponential model [95% CI]	d, intercept of linear model [95% CI]	Posterior probability for exponential model	Posterior probability for linear model	Bayes Factor in favour of exponential model	Bayes Factor in favour of difference in effect sizes between high and low moral incongruence participants
Craving for sexual intercourse	*Post-episode*	*Group B*	*Yes*	*High moral incongruence*	3.949 [2.384, 4.921]	0.635 [0.2401, 1.329]	1.047 [0.3305, 2.16]	1.811 [0.8464, 2.849]	1	8.138e-09	116900000	0.3914
				Low moral incongruence	1.524 [-4.321, 4.892]	5.764 [1.115, 9.814]	2.905 [1.49, 4.4]	2.919 [1.542, 4.312]	0.3637	0.6363	0.586	
			No	*High moral incongruence*	3.511 [2.091, 4.941]	0.9913 [0.2703, 2.512]	2.643 [0.6839, 5.694]	1.985 [0.96, 3.03]	1	1.271e-13	2.188e+14	0.1535
				Low moral incongruence	1.486 [-2.45, 3.846]	1.061 [0.007888, 8.676]	1.59 [0.4129, 2.761]	2.106 [1.101, 3.068]	0.2895	0.7105	0.3815	
		Group A	*Yes*	*High moral incongruence*	4.002 [2.355, 4.948]	0.5847 [0.2027, 1.277]	1.016 [0.3296, 2.049]	1.831 [0.8017, 2.901]	1	4.243e-09	209400000	0.3609
				Low moral incongruence	-0.982 [-4.547, 3.635]	6.18 [0.1865, 9.821]	2.744 [1.275, 4.055]	2.757 [1.555, 3.978]	0.1553	0.8447	0.1952	
			No	*High moral incongruence*	-0.04333 [-3.245, 3.426]	-1.474 [-5.067, -0.06341]	2.493 [1.105, 4.152]	2.545 [1.359, 3.851]	2.1e-07	1	1.063e-07	0.9239
				Low moral incongruence	-1.811 [-4.532, 2.366]	-6.397 [-9.838, -1.856]	2.937 [0.881, 4.432]	2.81 [1.369, 4.242]	0.9219	0.07808	12.27	
		Group B	*Yes*	*High moral incongruence*	2.288 [0.46, 4.233]	-0.31 [-1.678, -0.01492]	0.8841 [0.04326, 2.582]	1.988 [0.6583, 3.386]	0.8892	0.1108	4.932	0.3513
				Low moral incongruence	-0.9317 [-4.611, 3.8]	-5.142 [-9.634, -0.4875]	2.88 [0.9129, 5.09]	2.381 [0.7712, 4.067]	0.9971	0.002924	395.5	
			No	*High moral incongruence*	1.591 [-0.2503, 3.82]	-0.8633 [-4.108, -0.03665]	2.213 [0.6171, 3.695]	2.48 [1.164, 3.771]	0.934	0.066	8.307	1.094
				Low moral incongruence	-1.618 [-4.114, 1.052]	-6.468 [-9.797, -1.282]	3.282 [1.895, 4.913]	3.084 [1.65, 4.622]	0.5502	0.4498	1.233	

State variable	Pre- or post-episode	Type of sexual episode	Truncation	Moral incongruence level	a, amplitude of exponential model [95% CI]	b, decay constant of exponential model [95% CI]	c, offset of exponential model [95% CI]	d, intercept of linear model [95% CI]	Posterior probability for exponential model	Posterior probability for linear model	Bayes Factor in favour of exponential model	Bayes Factor in favour of difference in effect sizes between high and low moral incongruence participants
			Yes	*High moral incongruence*	2.279 [0.3761, 4.139]	-0.1666 [-0.6833, -0.01419]	0.8457 [0.03481, 2.36]	1.981 [0.7283, 3.32]	0.7692	0.2308	5.15	0.7086
				Low moral incongruence	-1.419 [-4.279, 1.715]	-6.522 [-9.801, -1.155]	2.788 [1.209, 4.553]	2.596 [1.215, 4.064]	0.405	0.595	0.5815	
	Pre-episode	*Group A*	*No*	*High moral incongruence*	2.403 [-0.04261, 4.784]	1.187 [0.1546, 2.364]	3.489 [0.7259, 8.471]	2.541 [1.189, 4.01]	2.195e-23	1	3.644e-19	
				Low moral incongruence	0.08784 [-2.974, 3.797]	1.558 [0.06517, 7.063]	3.214 [1.111, 4.209]	2.622 [1.418, 3.838]	0	1	0	
			Yes	*High moral incongruence*	2.926 [1.012, 4.236]	1.165 [0.3032, 2.63]	2.215 [0.6741, 3.309]	2.223 [0.8369, 3.687]	0.01005	0.9899	0.01919	
				Low moral incongruence	0.1787 [-4.651, 4.742]	6.077 [0.2797, 9.86]	3.092 [1.3, 4.862]	3.064 [1.299, 4.948]	0.2491	0.7509	0.3148	
		Group B	*No*	*High moral incongruence*	3.303 [1.32, 4.773]	0.5899 [0.1466, 1.603]	1.853 [0.7183, 3.141]	2.587 [1.141, 4.046]	1	3.733e-12	3.031e+11	0.1558
				Low moral incongruence	1.138 [-2.106, 3.81]	1.417 [0.02244, 5.266]	2.308 [1.001, 3.431]	2.795 [1.738, 3.942]	3.503e-05	1	1.803e-05	
			Yes	*High moral incongruence*	2.307 [0.8277, 4.79]	1.405 [0.2758, 3.015]	3.407 [0.6471, 8.162]	2.177 [0.8316, 3.59]	8.857e-06	1	3.63e-07	
				Low moral incongruence	1.96 [-2.894, 4.421]	4.91 [0.0279, 9.261]	3.715 [0.848, 6.046]	3.155 [1.819, 4.601]	0	1	0	
Difficulty thinking	*Post-episode*	*Group A*	*No*	*High moral incongruence*	1.174 [-1.176, 3.383]	-2.41 [-9.134, -0.09077]	2.748 [1.764, 3.648]	2.944 [2.112, 3.802]	0.9999	0.0001435	7378	0.4834
				Low moral incongruence	0.8832 [-1.567, 3.315]	-5.831 [-9.813, -1.211]	4.094 [3.243, 4.963]	4.129 [3.257, 5.053]	0.04748	0.9525	0.04968	
			Yes	*High moral incongruence*	1.859 [-0.3057, 3.582]	-2.997 [-9.561, -0.03273]	2.766 [1.018, 3.846]	3.131 [2.056, 4.172]	5.604e-11	1	1.611e-09	

State variable	Pre- or post-episode	Type of sexual episode	Truncation	Moral incongruence level	a, amplitude of exponential model [95% CI]	b, decay constant of exponential model [95% CI]	c, offset of exponential model [95% CI]	d, intercept of linear model [95% CI]	Posterior probability for exponential model	Posterior probability for linear model	Bayes Factor in favour of exponential model	Bayes Factor in favour of difference in effect sizes between high and low moral incongruence participants
				Low moral incongruence	1.377 [-1.484, 3.536]	-5.741 [-9.722, -1.277]	3.999 [2.938, 5.323]	4.437 [3.466, 5.478]	0.01875	0.9812	0.01446	
		Group B	*No*	*High moral incongruence*	1.22 [-0.946, 3.167]	-0.1833 [-0.4919, -0.03434]	2.353 [1.082, 3.4]	2.907 [2.084, 3.718]	1	5.257e-06	111700	0.2480
				Low moral incongruence	2.376 [-0.1857, 4.71]	-3.737 [-9.153, -0.6846]	3.952 [3.106, 4.872]	4.135 [3.338, 4.975]	0.9985	0.001471	597.6	
			Yes	*High moral incongruence*	1.618 [-0.523, 3.82]	-2.841 [-9.539, -0.02713]	2.638 [0.7833, 3.94]	3.178 [2.21, 4.172]	0.9422	0.05782	16.32	0.4189
				Low moral incongruence	2.651 [0.3713, 4.839]	-4.035 [-9.681, -0.7493]	3.888 [2.825, 5.083]	4.294 [3.441, 5.307]	0.9546	0.04545	17.27	
	Pre-episode	*Group A*	*No*	*High moral incongruence*	0.7014 [-2.68, 3.675]	7.28 [2.595, 9.881]	2.986 [2.003, 3.997]	2.96 [1.959, 3.915]	0.1296	0.8704	0.1641	
				Low moral incongruence	1.657 [-3.695, 4.79]	6.845 [1.769, 9.811]	4.562 [2.655, 7.237]	3.711 [2.695, 4.758]	0.01222	0.9878	0.0125	
			Yes	*High moral incongruence*	1.278 [-2.569, 3.683]	6.168 [2.193, 9.835]	2.974 [1.674, 3.89]	2.846 [1.747, 3.967]	0.002349	0.9977	0.002039	
				Low moral incongruence	1.173 [-4.365, 4.871]	5.544 [1.131, 9.804]	3.463 [2.416, 4.575]	3.472 [2.382, 4.61]	0.3569	0.6431	0.5598	
		Group B	*No*	*High moral incongruence*	0.9826 [-2.47, 3.446]	7.177 [2.541, 9.88]	2.975 [2.003, 3.988]	2.918 [1.94, 3.85]	0.1627	0.8373	0.1791	
				Low moral incongruence	1.387 [-2.978, 4.684]	6.535 [1.777, 9.862]	3.766 [2.884, 4.513]	3.794 [3.024, 4.554]	0.1253	0.8747	0.1645	
			Yes	*High moral incongruence*	0.5007 [-2.669, 3.076]	6.784 [1.876, 9.905]	2.839 [1.603, 3.998]	2.799 [1.608, 3.841]	0.05699	0.943	0.06393	
				Low moral incongruence	1.725 [-2.549, 4.681]	6.084 [1.194, 9.776]	3.47 [2.649, 4.341]	3.494 [2.819, 4.223]	0.1892	0.8108	0.2621	

State variable	Pre- or post-episode	Type of sexual episode	Truncation	Moral incongruence level	a, amplitude of exponential model [95% CI]	b, decay constant of exponential model [95% CI]	c, offset of exponential model [95% CI]	d, intercept of linear model [95% CI]	Posterior probability for exponential model	Posterior probability for linear model	Bayes Factor in favour of exponential model	Bayes Factor in favour of difference in effect sizes between high and low moral incongruence participants
Guilt	*Post-episode*	*Group A*	*No*	*High moral incongruence*	1.569 [-1.841, 4.44]	-0.06303 [-0.1019, -0.03525]	2.58 [0.8912, 4.341]	3.27 [2.091, 4.601]	1	2.764e-23	3.673e+22	0.1484
				Low moral incongruence	0.5651 [-2.619, 3.631]	-0.8065 [-3.535, -0.1047]	3.743 [1.709, 5.593]	3.977 [2.119, 5.808]	0.9999	6.77e-05	8293	
			Yes	*High moral incongruence*	3.172 [1.311, 4.87]	-0.2932 [-0.9568, -0.02471]	2.325 [0.5316, 4.005]	3.773 [2.392, 5.202]	1	1.201e-06	2429000	0.1937
				Low moral incongruence	-1.128 [-3.665, 2.179]	-3.107 [-7.108, -0.1352]	5.897 [1.416, 8.11]	4.248 [1.88, 6.702]	0	1	0	
		Group B	*No*	*High moral incongruence*	1.461 [-1.867, 4.161]	-0.07192 [-0.1524, -0.03778]	2.628 [0.927, 4.279]	3.187 [2.053, 4.255]	1	2.731e-27	3.387e+26	0.1630
				Low moral incongruence	0.8886 [-1.89, 3.323]	-0.5887 [-2.152, -0.1013]	3.914 [1.995, 5.8]	4.22 [2.405, 5.957]	1	5.983e-08	17630000	
			Yes	*High moral incongruence*	2.491 [0.0793, 4.808]	-1.335 [-4.654, -0.02141]	2.018 [0.445, 3.991]	3.784 [2.396, 5.24]	2.828e-15	1	3.489e-16	0.1471
				Low moral incongruence	-0.1143 [-3.219, 3.087]	-0.398 [-1.562, -0.08873]	4.457 [1.912, 7.175]	4.344 [2.035, 6.553]	0.9994	0.0005635	1997	
	Pre-episode	*Group A*	*No*	*High moral incongruence*	0.8577 [-2.9, 4.313]	5.932 [1.965, 9.849]	3.079 [2.069, 4.511]	3.255 [2.075, 4.482]	0.0004706	0.9995	0.0005057	
				Low moral incongruence	0.9901 [-3.798, 4.732]	6.622 [1.841, 9.865]	3.743 [1.782, 5.639]	3.798 [2.021, 5.555]	0.1857	0.8143	0.2142	
			Yes	*High moral incongruence*	3.165 [0.6806, 4.862]	2.038 [0.5881, 6.368]	2.273 [0.7303, 3.818]	2.458 [0.936, 3.978]	0.9998	0.0002365	4639	0.6912
				Low moral incongruence	0.8329 [-4.556, 4.81]	6.074 [1.072, 9.852]	3.446 [1.548, 5.316]	3.529 [1.859, 5.272]	0.2739	0.7261	0.3839	

**State variable**	**Pre- or post-episode**	**Type of sexual episode**	**Truncation**	**Moral incongruence level**	**a, amplitude of exponential model [95% CI]**	**b, decay constant of exponential model [95% CI]**	**c, offset of exponential model [95% CI]**	**d, intercept of linear model [95% CI]**	**Posterior probability for exponential model**	**Posterior probability for linear model**	**Bayes Factor in favour of exponential model**	**Bayes Factor in favour of difference in effect sizes between high and low moral incongruence participants**
		Group B	*No*	*High moral incongruence*	2.129 [-0.441, 4.482]	7.033 [2.769, 9.885]	3.287 [2.059, 4.616]	3.287 [1.993, 4.692]	0.6805	0.3195	2.058	0.1886
				Low moral incongruence	-0.3942 [-3.322, 3.29]	5.249 [1.551, 9.801]	4.152 [2.448, 5.855]	3.944 [2.181, 5.605]	0.001481	0.9985	0.001649	
			Yes	*High moral incongruence*	1.71 [-2.478, 4.824]	2.104 [0.5797, 6.427]	2.717 [0.9603, 3.898]	2.492 [1.061, 4.032]	3.208e-06	1	2.739e-07	
				Low moral incongruence	-0.1879 [-3.629, 3.508]	5.09 [1.094, 9.816]	4.544 [2.295, 6.404]	3.857 [2.042, 5.618]	0.0001046	0.9999	0.0004216	
Loneliness	*Post-episode*	*Group A*	*No*	*High moral incongruence*	2.405 [0.6996, 3.291]	-1.279 [-3.267, -0.07457]	2.994 [0.7855, 5.491]	3.676 [2.201, 5.081]	3.923e-07	1	2.27e-08	
				Low moral incongruence	-0.1548 [-2.725, 2.496]	-5.474 [-9.62, -0.462]	5.099 [2.672, 7.226]	4.384 [2.524, 6.258]	0.00507	0.9949	0.0006835	
			Yes	*High moral incongruence*	0.6083 [-3.578, 3.178]	-1.491 [-2.702, -0.06056]	4.389 [2.13, 7.592]	4.139 [2.621, 5.705]	0	1	0	
				Low moral incongruence	0.1323 [-3.233, 3.102]	-6.319 [-9.842, -1.119]	4.439 [2.046, 6.698]	4.366 [2.362, 6.381]	0.2023	0.7977	0.2808	
		Group B	*No*	*High moral incongruence*	0.4932 [-3.6, 3.433]	-0.5259 [-1.605, -0.02977]	3.882 [1.431, 6.257]	3.593 [2.234, 5.052]	3.036e-35	1	1.438e-33	
				Low moral incongruence	0.5301 [-1.709, 2.256]	-5.561 [-9.762, -0.6401]	4.822 [2.948, 6.882]	4.736 [2.901, 6.462]	0.2078	0.7922	0.2822	
			Yes	*High moral incongruence*	1.775 [0.2911, 3.562]	-1.139 [-7.489, -0.043]	3.531 [1.655, 5.298]	4.117 [2.54, 5.713]	0.9794	0.02063	38.02	12956
				Low moral incongruence	-0.0926 [-2.791, 1.987]	-6.4 [-9.826, -1.351]	4.792 [2.508, 7.078]	4.635 [2.44, 6.707]	0.08023	0.9198	0.07489	
	Pre-episode	*Group A*	*No*	*High moral incongruence*	0.6929 [-1.303, 3.474]	6.335 [2.078, 9.78]	4.082 [2.449, 5.543]	3.82 [2.38, 5.264]	5.491e-09	1	3.513e-10	

State variable	Pre- or post-episode	Type of sexual episode	Truncation	Moral incongruence level	a, amplitude of exponential model [95% CI]	b, decay constant of exponential model [95% CI]	c, offset of exponential model [95% CI]	d, intercept of linear model [95% CI]	Posterior probability for exponential model	Posterior probability for linear model	Bayes Factor in favour of exponential model	Bayes Factor in favour of difference in effect sizes between high and low moral incongruence participants
				Low moral incongruence	0.326 [-4.36, 4.67]	6.578 [1.841, 9.85]	4.113 [2.099, 6.137]	4.127 [2.319, 6.087]	0.1866	0.8134	0.2219	
			Yes	*High moral incongruence*	2.114 [-0.5508, 4.409]	6.252 [1.425, 9.846]	3.532 [1.894, 5.375]	3.638 [1.962, 5.514]	0.5429	0.4571	1.285	1.350
				Low moral incongruence	1.815 [-4.106, 4.79]	4.887 [1.689, 9.753]	5.201 [1.999, 8.132]	4.13 [2.045, 6.01]	2.836e-11	1	1.34e-09	
		Group B	*No*	*High moral incongruence*	1.244 [-1.278, 3.628]	6.661 [1.778, 9.878]	3.734 [2.145, 5.37]	3.75 [2.146, 5.254]	0.1202	0.8798	0.1231	
				Low moral incongruence	-0.03956 [-4.211, 3.507]	5.243 [1.372, 9.8]	4.628 [2.678, 6.439]	4.31 [2.534, 6.214]	2.909e-18	1	2.211e-21	
			Yes	*High moral incongruence*	2.126 [-0.3859, 4.214]	5.969 [1.325, 9.821]	3.5 [1.738, 5.237]	3.663 [2.028, 5.397]	0.6606	0.3394	2.026	139.1
				Low moral incongruence	-1.159 [-4.169, 3.216]	6.954 [1.559, 9.856]	5.408 [2.798, 7.374]	4.458 [2.315, 6.538]	2.976e-08	1	1.037e-09	
Mood	*Post-episode*	*Group A*	*No*	*High moral incongruence*	-0.4816 [-4.256, 3.488]	-5.485 [-9.642, -1.379]	4.414 [2.057, 5.571]	5.151 [4.754, 5.593]	0.1926	0.8074	4.187	0.3885
				Low moral incongruence	-0.805 [-2.504, 1.385]	-4.871 [-9.619, -0.6949]	4.957 [4.116, 5.792]	4.911 [4.164, 5.682]	0.2798	0.7202	0.4003	
			Yes	*High moral incongruence*	-1.744 [-3.214, -0.2026]	-0.4951 [-2.019, -0.09803]	5.531 [4.87, 6.339]	5.062 [4.426, 5.694]	1	8.433e-06	123200	0.1742
				Low moral incongruence	-0.8137 [-2.673, 1.204]	-5.247 [-9.71, -0.317]	4.893 [3.506, 6.232]	4.811 [3.702, 5.849]	0.0937	0.9063	0.1046	
		Group B	*No*	*High moral incongruence*	-1.879 [-4.302, 0.2268]	-5.643 [-9.523, -1.328]	5.271 [4.846, 5.662]	5.202 [4.681, 5.749]	1	2.97e-05	35950	0.1965
				Low moral incongruence	-0.8593 [-1.983, 0.5848]	-5.084 [-9.625, -0.8834]	4.882 [4.085, 5.684]	4.851 [4.169, 5.557]	0.3506	0.6494	0.5658	

State variable	Pre- or post-episode	Type of sexual episode	Truncation	Moral incongruence level	a, amplitude of exponential model [95% CI]	b, decay constant of exponential model [95% CI]	c, offset of exponential model [95% CI]	d, intercept of linear model [95% CI]	Posterior probability for exponential model	Posterior probability for linear model	Bayes Factor in favour of exponential model	Bayes Factor in favour of difference in effect sizes between high and low moral incongruence participants
			Yes	*High moral incongruence*	-1.656 [-3.458, -0.1841]	-0.576 [-2.944, -0.1021]	5.502 [4.793, 6.31]	5.053 [4.441, 5.663]	1	3.633e-06	319300	1.124
				Low moral incongruence	-0.685 [-1.849, 0.7737]	-4.153 [-9.621, -0.4769]	4.144 [2.106, 5.779]	4.762 [3.77, 5.693]	0.0004681	0.9995	0.0001069	
	Pre-episode	*Group A*	*No*	*High moral incongruence*	-0.654 [-3.126, 1.505]	6.293 [2.678, 9.686]	4.877 [3.216, 6.004]	5.143 [4.659, 5.633]	0	1	0	
				Low moral incongruence	-1.621 [-4.796, 3.557]	6.809 [2.072, 9.874]	5.042 [4.174, 5.913]	5.033 [4.218, 5.823]	0.1888	0.8112	0.2247	
			Yes	*High moral incongruence*	-0.1133 [-2.694, 1.869]	5.69 [1.262, 9.664]	5.345 [4.828, 5.677]	5.173 [4.739, 5.624]	1.364e-05	1	6.025e-06	
				Low moral incongruence	-0.08805 [-4.677, 4.63]	6.691 [1.736, 9.916]	5.201 [3.848, 6.476]	5.204 [4.084, 6.285]	0.1474	0.8526	0.1807	
		Group B	*No*	*High moral incongruence*	-0.3179 [-2.304, 1.471]	5.577 [1.979, 9.799]	5.683 [4.659, 7.271]	5.144 [4.616, 5.639]	1.558e-06	1	2.264e-06	
				Low moral incongruence	0.9979 [-2.487, 4.044]	6.82 [2.141, 9.819]	5.089 [4.287, 5.945]	5.047 [4.202, 5.842]	0.06449	0.9355	0.06895	
			Yes	*High moral incongruence*	-0.1504 [-2.245, 3.52]	4.557 [1.528, 9.686]	5.669 [4.574, 7.357]	5.162 [4.751, 5.639]	7.276e-08	1	1.076e-07	
				Low moral incongruence	1.653 [-2.001, 4.506]	6.857 [2.17, 9.865]	5.331 [4.321, 6.29]	5.386 [4.419, 6.319]	0.1363	0.8637	0.1601	
Relationship connectedness	*Post-episode*	*Group A*	*No*	*High moral incongruence*	-1.519 [-2.51, -0.5832]	-0.3044 [-1.18, -0.07643]	4.946 [3.782, 6.103]	4.597 [3.464, 5.709]	1	1.111e-05	86530	1.360
				Low moral incongruence	0.02974 [-2.316, 2.188]	-5.037 [-9.792, -1.478]	4.216 [2.373, 6.489]	4.311 [2.311, 6.271]	5.291e-06	1	2.087e-05	
			Yes	*High moral incongruence*	-1.547 [-2.834, -0.7272]	-0.4744 [-3.969, -0.02671]	5.21 [3.684, 6.903]	4.531 [3.193, 5.783]	0.9784	0.0216	40.81	13.97

State variable	Pre- or post-episode	Type of sexual episode	Truncation	Moral incongruence level	a, amplitude of exponential model [95% CI]	b, decay constant of exponential model [95% CI]	c, offset of exponential model [95% CI]	d, intercept of linear model [95% CI]	Posterior probability for exponential model	Posterior probability for linear model	Bayes Factor in favour of exponential model	Bayes Factor in favour of difference in effect sizes between high and low moral incongruence participants
				Low moral incongruence	-0.4163 [-2.342, 1.724]	-6.368 [-9.835, -1.392]	4.432 [2.033, 7.12]	4.346 [2.011, 6.686]	0.0295	0.9705	0.03299	
		Group B	*No*	*High moral incongruence*	-1.574 [-2.732, -0.6976]	-0.3052 [-1.037, -0.08938]	4.915 [3.736, 6.205]	4.59 [3.497, 5.661]	1	2.269e-06	562200	112.6
				Low moral incongruence	-0.3583 [-1.593, 0.9636]	-6.413 [-9.864, -1.554]	4.175 [2.111, 6.054]	4.239 [2.126, 6.355]	0.01006	0.9899	0.01004	
			Yes	*High moral incongruence*	-1.528 [-2.785, -0.7573]	-0.5578 [-4.33, -0.03532]	5.03 [3.901, 6.677]	4.486 [3.237, 5.78]	0.6511	0.3489	2.233	3853
				Low moral incongruence	-0.5325 [-2.088, 1.073]	-6.475 [-9.856, -1.574]	4.267 [1.827, 6.747]	4.275 [1.964, 6.591]	0.01875	0.9813	0.01991	
	Pre-episode	*Group A*	*No*	*High moral incongruence*	-0.8149 [-2.929, 1.747]	7.081 [2.319, 9.929]	4.468 [3.115, 5.627]	4.46 [3.25, 5.701]	0.07009	0.9299	0.08506	
				Low moral incongruence	-1.043 [-4.76, 4.124]	7.012 [2.125, 9.899]	4.172 [1.753, 6.412]	4.348 [2.005, 6.741]	0.1857	0.8143	0.2398	
			Yes	*High moral incongruence*	-1.232 [-3.425, 0.858]	6.742 [1.97, 9.893]	4.783 [3.398, 6.178]	4.688 [3.474, 5.899]	0.1527	0.8473	0.1786	
				Low moral incongruence	0.08245 [-4.749, 4.737]	6.947 [2.221, 9.887]	4.311 [0.983, 7.607]	4.402 [1.684, 7.323]	0.1883	0.8117	0.2529	
		Group B	*No*	*High moral incongruence*	0.324 [-2.717, 3.801]	6.435 [2.522, 9.828]	5.199 [3.228, 7.326]	4.496 [3.285, 5.62]	3.918e-14	1	1.359e-16	
				Low moral incongruence	-0.1123 [-3.892, 4.025]	6.197 [2.019, 9.786]	4.127 [2.038, 6.292]	4.221 [1.984, 6.448]	2.465e-14	1	2.914e-13	
			Yes	*High moral incongruence*	-1.772 [-3.381, 0.6164]	5.508 [1.33, 9.792]	4.561 [3.527, 6.066]	4.663 [3.377, 5.923]	0.004951	0.995	0.004087	
				Low moral incongruence	-0.4637 [-4.119, 3.972]	6.589 [1.78, 9.869]	4.43 [1.731, 7.186]	4.438 [1.912, 7.041]	0.08912	0.9109	0.1022	

State variable	Pre- or post-episode	Type of sexual episode	Truncation	Moral incongruence level	a, amplitude of exponential model [95% CI]	b, decay constant of exponential model [95% CI]	c, offset of exponential model [95% CI]	d, intercept of linear model [95% CI]	Posterior probability for exponential model	Posterior probability for linear model	Bayes Factor in favour of exponential model	Bayes Factor in favour of difference in effect sizes between high and low moral incongruence participants
Shame	*Post-episode*	*Group A*	*No*	*High moral incongruence*	2.55 [-0.7324, 4.631]	-0.1765 [-0.5705, -0.03407]	2.243 [0.5988, 3.975]	3.3 [2.106, 4.6]	1	1.751e-29	1.12e+27	0.2511
				Low moral incongruence	1.564 [-0.7392, 3.537]	-4.403 [-9.241, -0.5658]	3.714 [1.848, 5.816]	3.854 [2.064, 5.658]	0.8908	0.1092	7.058	
			Yes	*High moral incongruence*	3.547 [1.503, 4.874]	-0.3405 [-1.1, -0.0357]	2.23 [0.4492, 4.09]	3.616 [2.067, 5.135]	1	2.376e-13	8.974e+12	0.1932
				Low moral incongruence	0.4662 [-3.595, 4.109]	-3.071 [-8.968, -0.1409]	4.264 [1.483, 7.123]	4.344 [2.06, 6.661]	0.9785	0.02146	72.92	
		Group B	*No*	*High moral incongruence*	2.372 [-0.3717, 4.519]	-0.2511 [-0.8192, -0.04674]	2.592 [0.9092, 4.263]	3.276 [2.009, 4.574]	1	1.743e-26	2.029e+26	0.3270
				Low moral incongruence	0.6865 [-2.457, 3.251]	-3.525 [-8.892, -0.419]	5.081 [2.01, 8.723]	4.047 [2.192, 5.788]	4.147e-16	1	1.3e-16	
			Yes	*High moral incongruence*	3.474 [1.526, 4.89]	-0.3348 [-0.9799, -0.04429]	2.307 [0.5846, 4.023]	3.638 [2.172, 5.167]	1	4.008e-13	3.535e+12	0.1662
				Low moral incongruence	0.6556 [-3.177, 3.809]	-2.126 [-7.913, -0.1404]	4.362 [1.726, 6.84]	4.412 [2.038, 6.413]	0.9981	0.001915	413.8	
	Pre-episode	*Group A*	*No*	*High moral incongruence*	2.338 [-0.0798, 4.464]	6.771 [2.456, 9.856]	3.266 [1.881, 4.649]	3.288 [2.028, 4.571]	0.8642	0.1358	7.386	0.8498
				Low moral incongruence	-0.6471 [-4.685, 4.457]	6.761 [1.767, 9.866]	3.87 [1.481, 6.188]	3.744 [1.799, 5.811]	0.163	0.837	0.1761	
			Yes	*High moral incongruence*	3.161 [0.7788, 4.746]	1.798 [0.4274, 4.172]	3.443 [0.8196, 6.89]	2.662 [1.144, 4.094]	3.082e-14	1	6.368e-15	
				Low moral incongruence	0.01284 [-4.681, 4.548]	5.39 [1.671, 9.85]	3.465 [1.919, 6.005]	3.841 [1.798, 5.81]	0.002912	0.9971	0.002984	

State variable	Pre- or post-episode	Type of sexual episode	Truncation	Moral incongruence level	a, amplitude of exponential model [95% CI]	b, decay constant of exponential model [95% CI]	c, offset of exponential model [95% CI]	d, intercept of linear model [95% CI]	Posterior probability for exponential model	Posterior probability for linear model	Bayes Factor in favour of exponential model	Bayes Factor in favour of difference in effect sizes between high and low moral incongruence participants
		Group B	*No*	*High moral incongruence*	2.418 [-0.4288, 4.592]	6.065 [1.895, 9.773]	3.262 [1.814, 4.69]	3.292 [1.902, 4.674]	0.9504	0.04959	15.98	0.2225
				Low moral incongruence	-0.07608 [-4.024, 3.614]	6.164 [0.408, 9.829]	3.956 [2.008, 6.026]	3.986 [2.063, 5.835]	0.08324	0.9168	0.08745	
			Yes	*High moral incongruence*	2.933 [0.4712, 4.851]	1.5 [0.4052, 4.736]	2.308 [0.7545, 3.879]	2.577 [1.209, 4.062]	1	7.775e-06	107000	0.2708
				Low moral incongruence	0.2865 [-3.597, 4.193]	6.283 [1.233, 9.882]	4.278 [2.05, 6.429]	4.109 [2.161, 6.056]	0.09579	0.9042	0.1115	

1.3 Graphs of temporal dynamics

Below are all graphs produced from fitting hierarchical exponential models to state variables obtained pre- and post-sexual episode via Bayesian modeling. Only the 24 hours before and after sexual episodes are displayed. Graphs on the left are for low moral incongruence participants, while graphs on the right are for high moral incongruence participants. Error ribbons represent 95% credible intervals. Exponential models are presented in cases where $BF_{10} > 1$, indicating evidence in favour of the alternative hypothesis, while horizontal models indicate cases where $BF_{01} > 1$, indicating evidence in favour of the null hypothesis.

Group A: any episodes of pornography use, either with or without masturbation or orgasm. Group B: any episodes of pornography use, either with or without masturbation or orgasm, plus episodes of masturbation without pornography use.

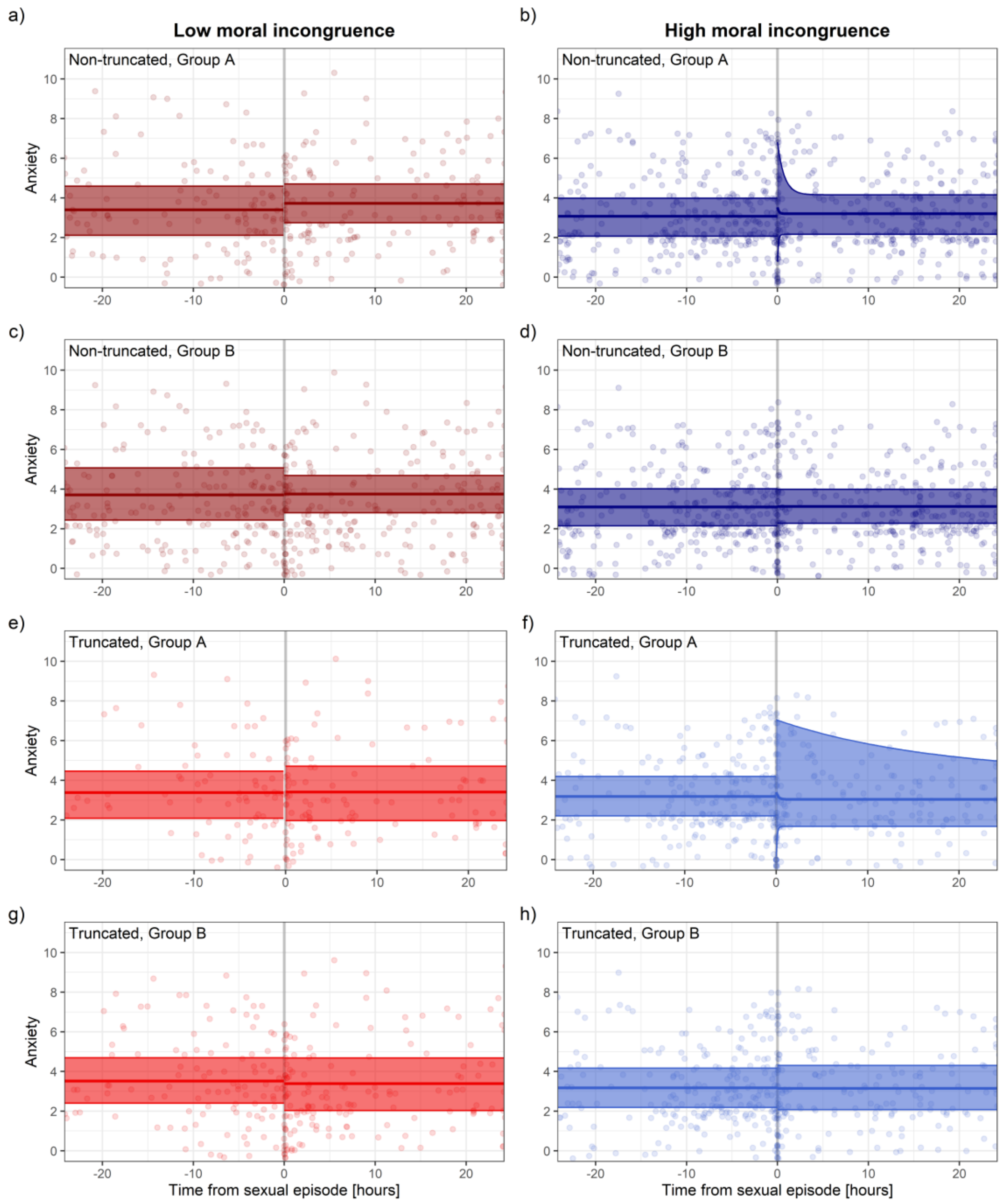

Figure A1: Results from fitting hierarchical exponential models to anxiety scores obtained pre- and post-sexual episodes.

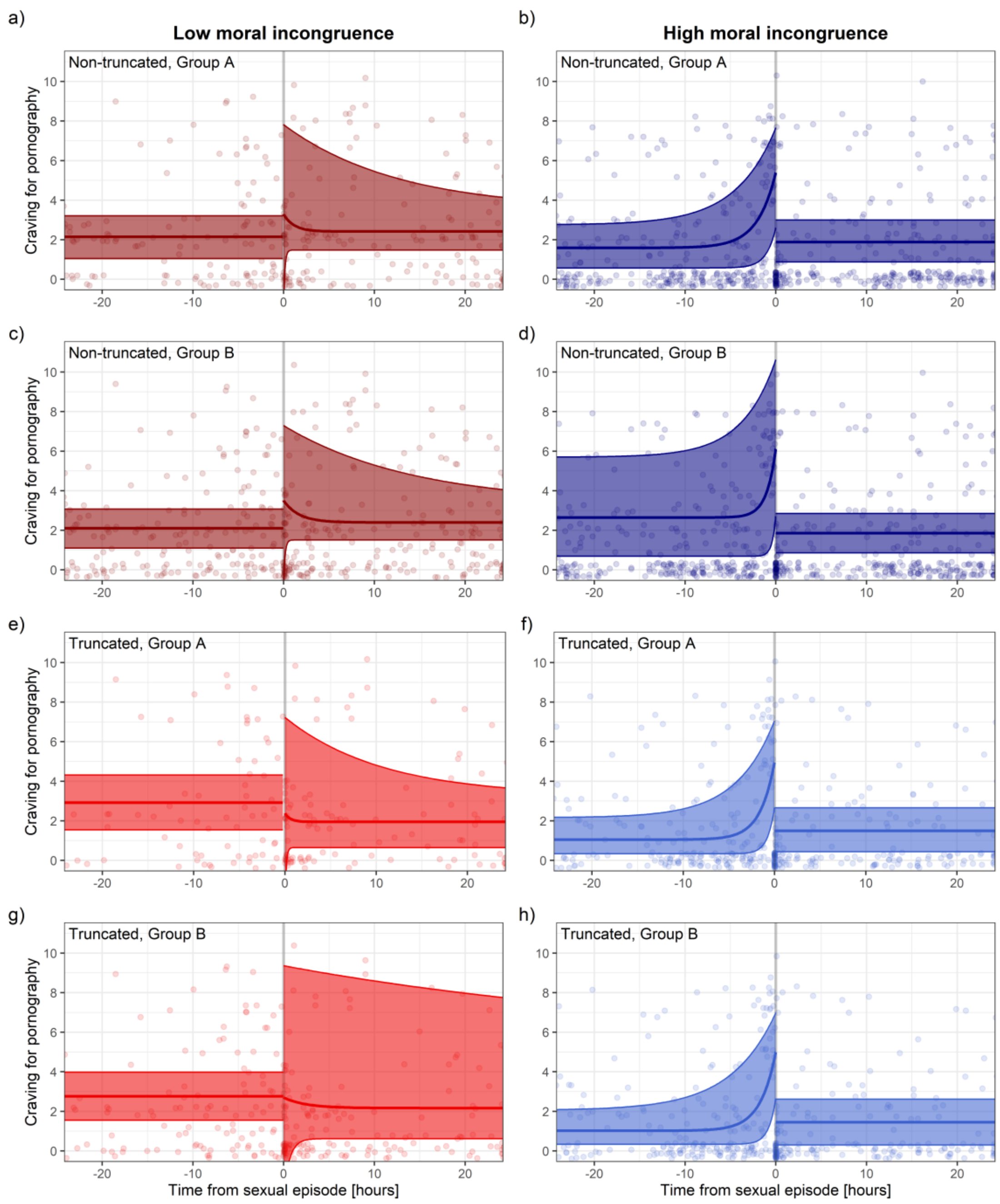

Figure A2: Results from fitting hierarchical exponential models to craving for pornography scores obtained pre- and post-sexual episodes.

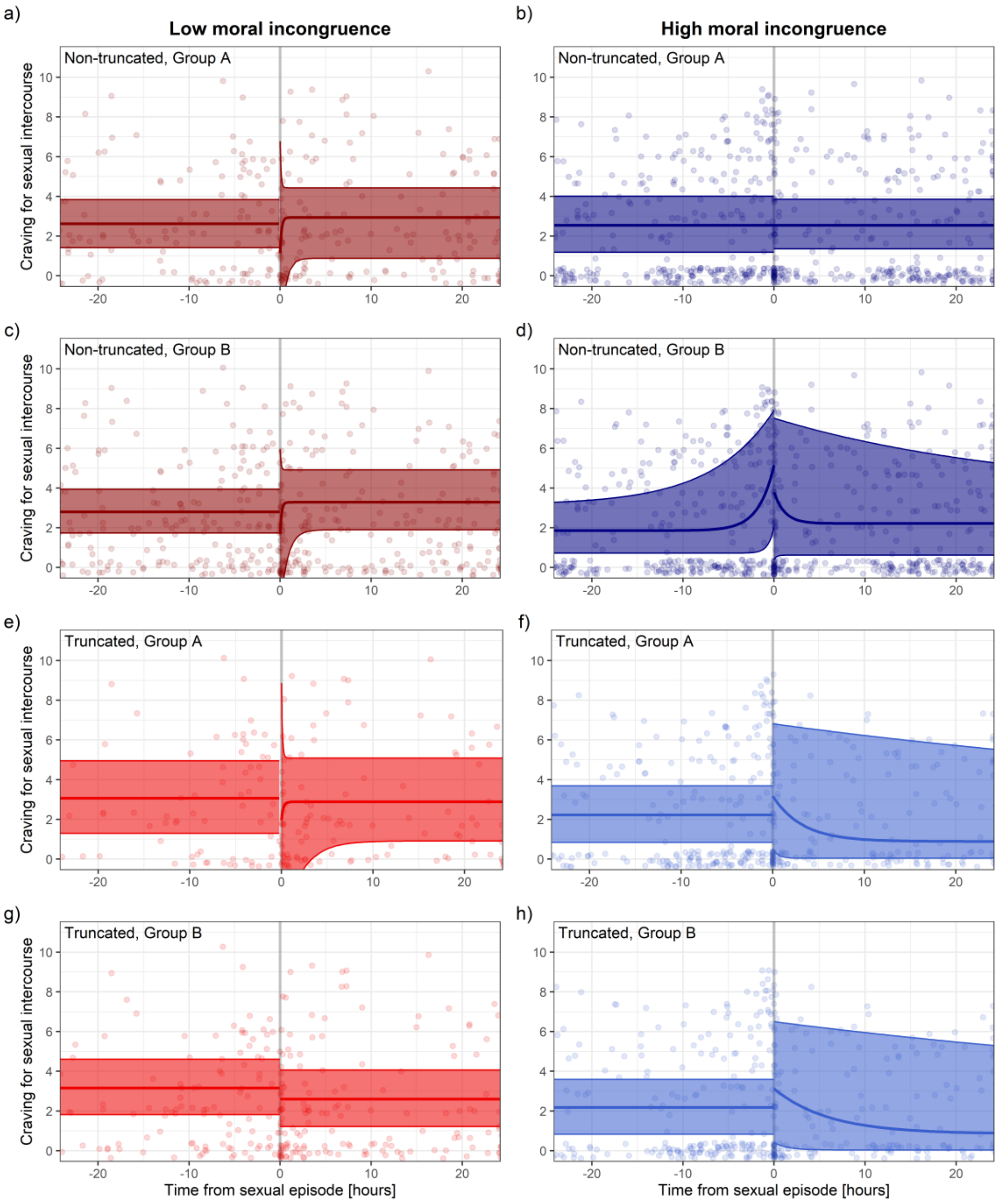

Figure A3: Results from fitting hierarchical exponential models to craving for sexual intercourse scores obtained pre- and post-sexual episodes.

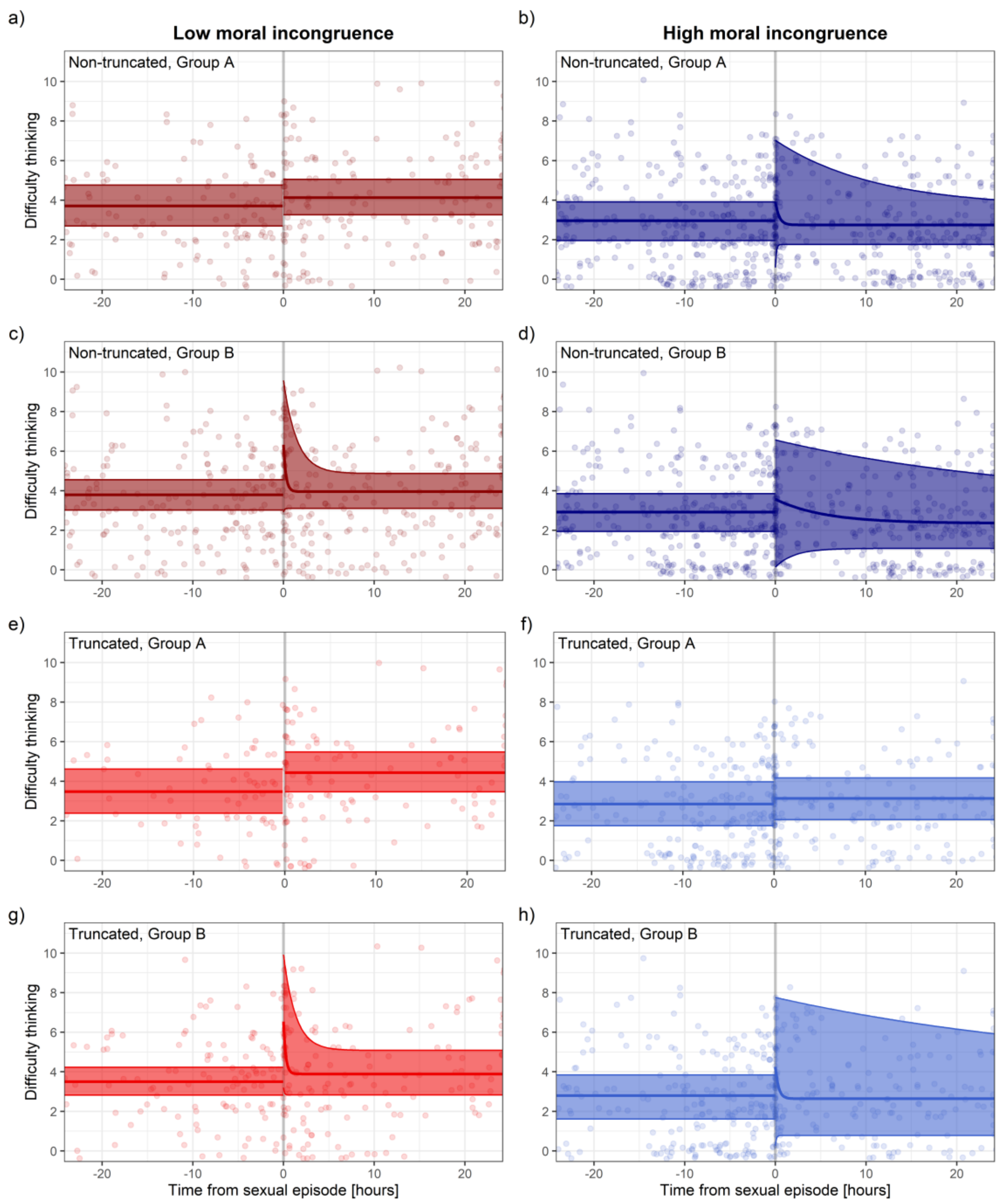

Figure A4: Results from fitting hierarchical exponential models to difficulty thinking scores obtained pre- and post-sexual episodes.

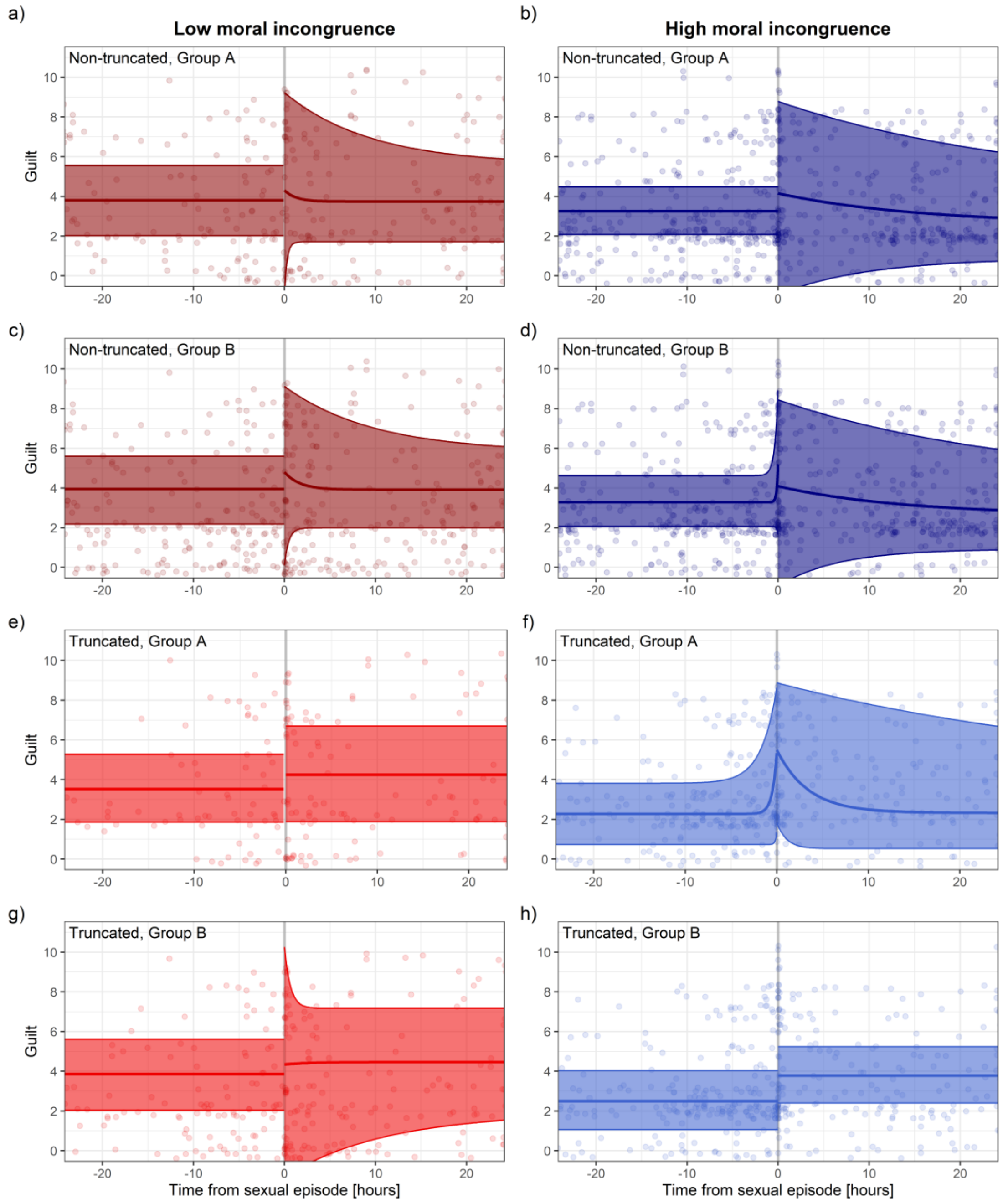

Figure A5: Results from fitting hierarchical exponential models to guilt scores obtained pre- and post-sexual episodes.

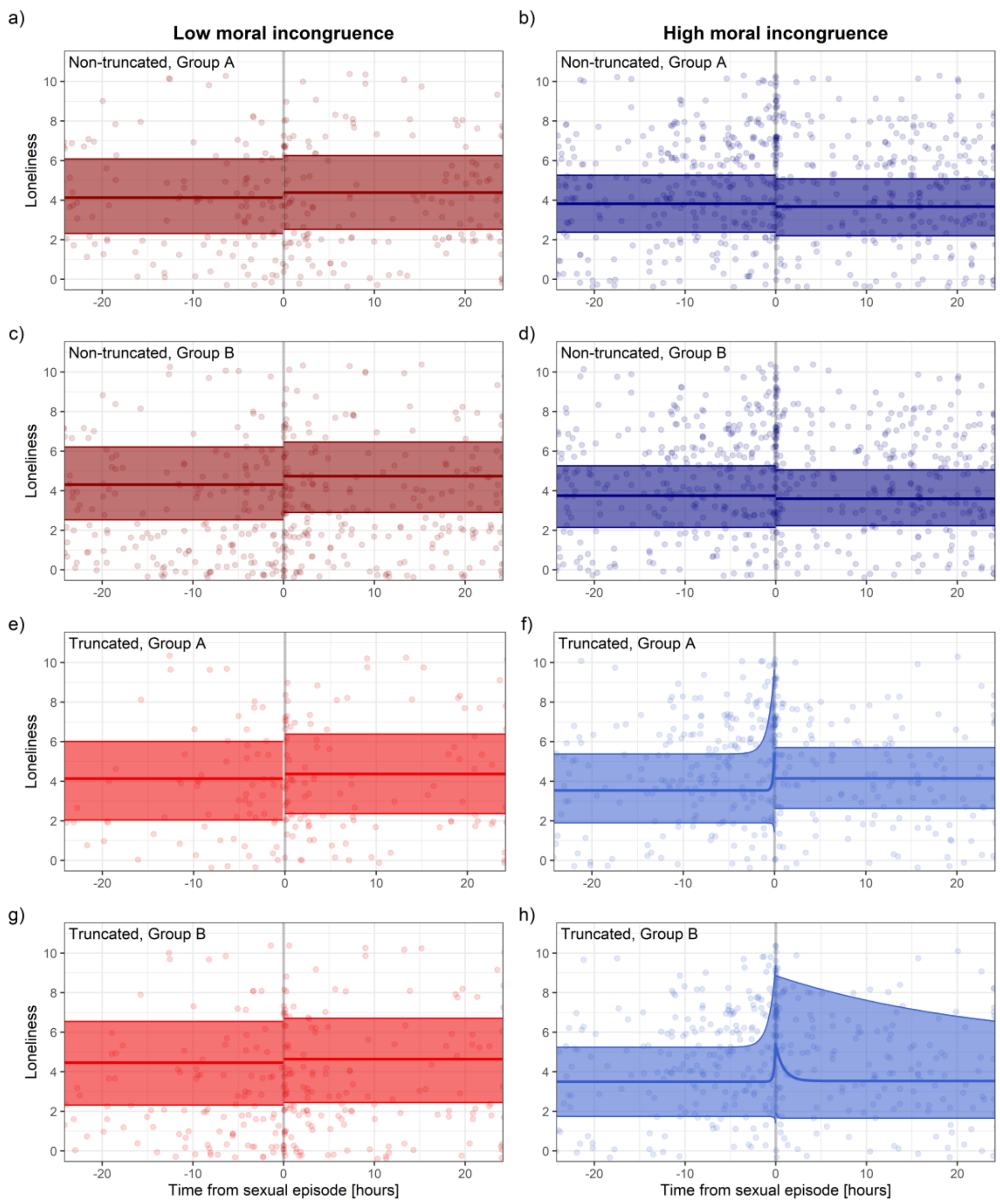

Figure A6: Results from fitting hierarchical exponential models to loneliness scores obtained pre- and post-sexual episodes.

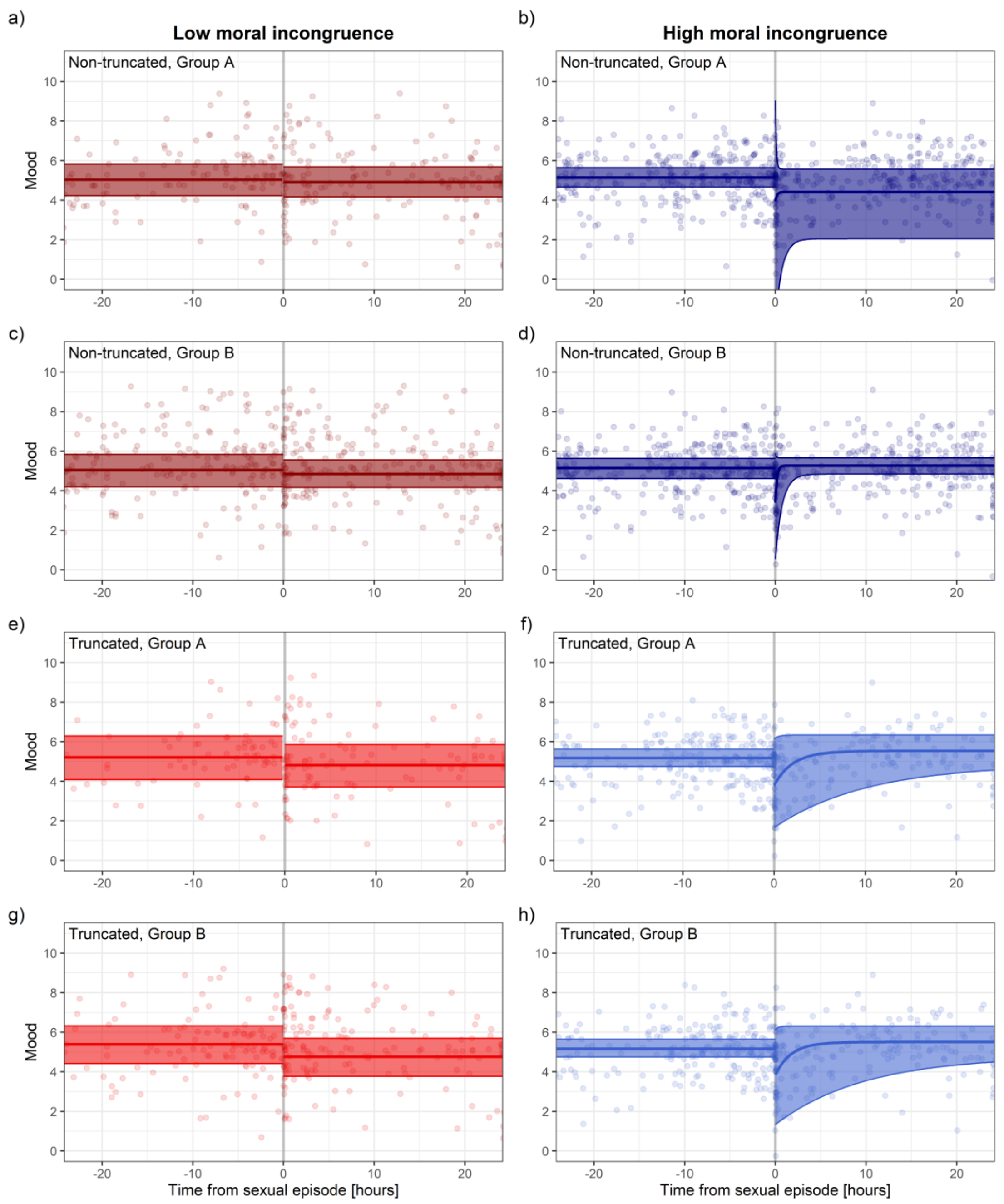

Figure A7: Results from fitting hierarchical exponential models to mood scores obtained pre- and post-sexual episodes.

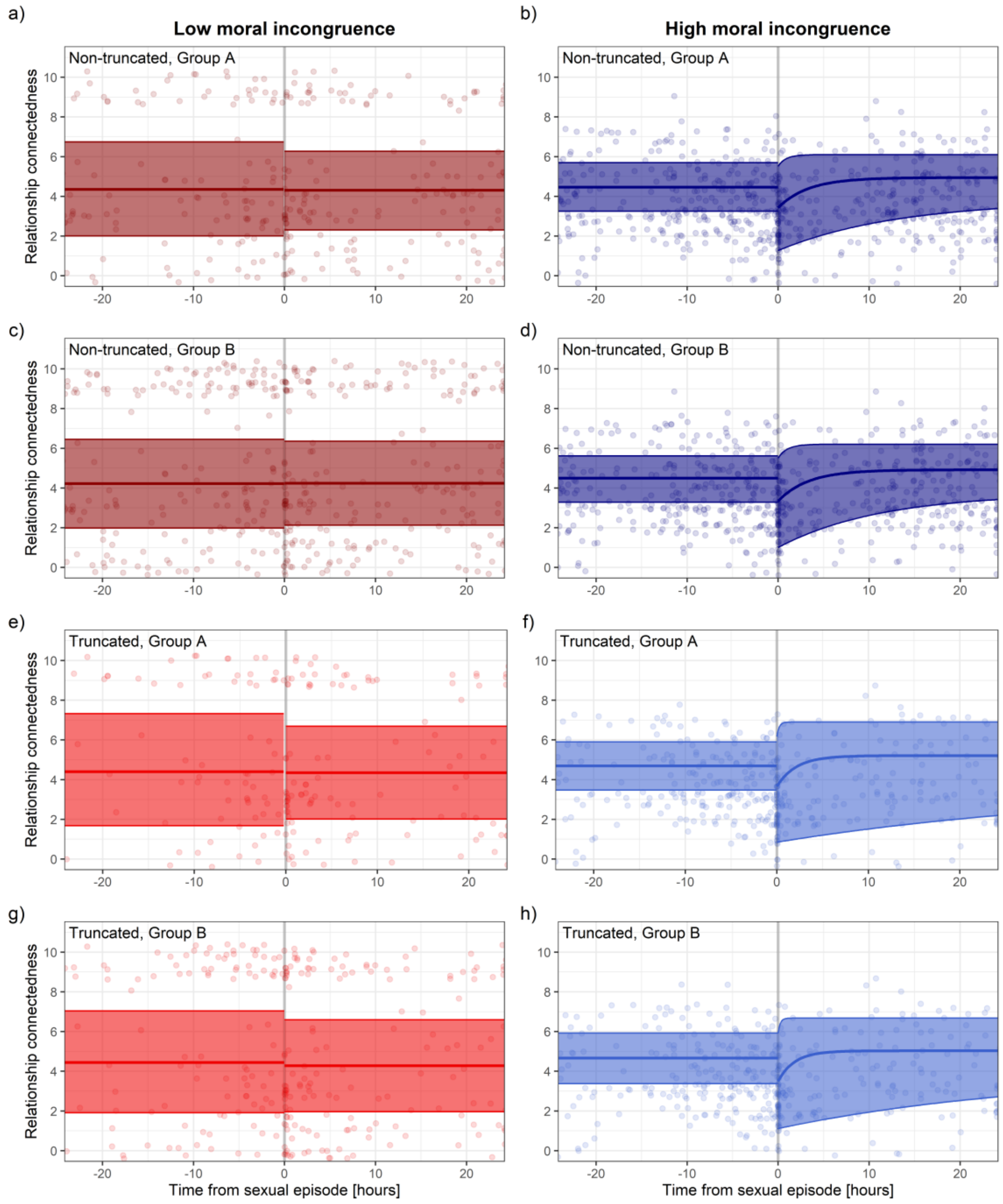

Figure A8: Results from fitting hierarchical exponential models to relationship connectedness scores obtained pre- and post-sexual episodes.

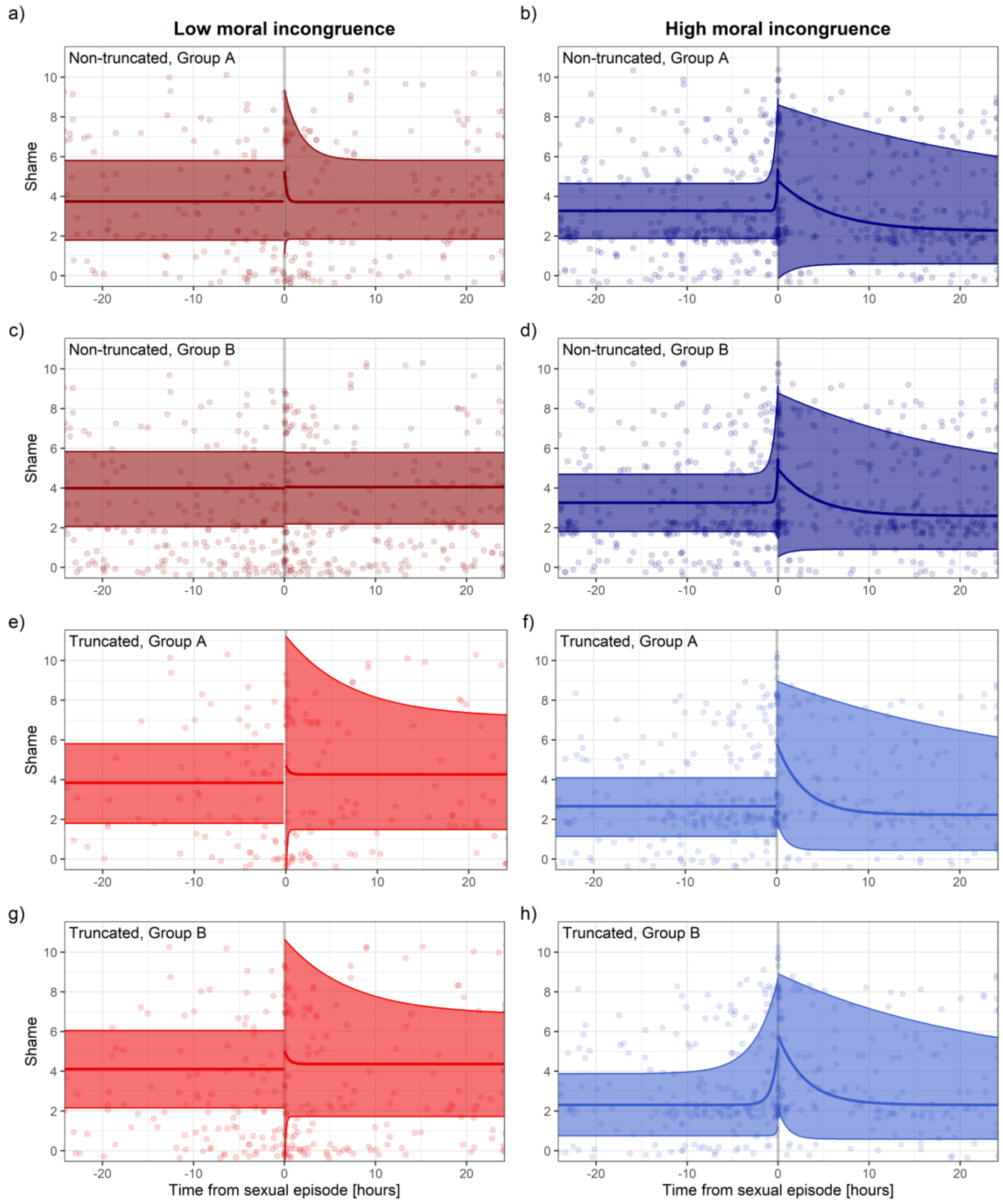

Figure A9: Results from fitting hierarchical exponential models to shame scores obtained pre- and post-sexual episodes.
