# Supplementary material for: Quantifying Opponent Process Dynamics in Pornography Use and Masturbation: An Exploratory Ecological Momentary Assessment Study
Source: Arch Sex Behav. 2025 Nov 21;54(9):3313–34. doi: 10.1007/s10508-025-03287-z (PMC12675693; doi:10.1007/s10508-025-03287-z)
Supplement: Supplementary file 2 — Supplementary file2 (PDF 75 KB) [file 10508_2025_3287_MOESM2_ESM.pdf]

Main survey

Please complete the survey below.

Thank you!

Below are some demographic questions for you to answer.

Which ethnic group (or groups) do you belong to? You may select multiple answers.

☐ New Zealand European ☐ Māori ☐ Samoan ☐ Cook Island Māori ☐ Tongan ☐ Niuean
☐ Chinese ☐ Indian ☐ Other (such as Dutch, Japanese, Tokelauan)

You selected 'other' as an option for your ethnic group. Which of these ethnic groups do you belong to? Please select all that apply

☐ English ☐ Australian ☐ Dutch ☐ Other European ☐ Tokelauan ☐ Fijian ☐ Other Pacific Peoples
☐ Filipino ☐ Japanese ☐ Korean ☐ Cambodian ☐ Other Asian ☐ Middle Eastern
☐ Latin American ☐ African ☐ Other

If other ethnicity, please specify: ______________________

What is your sex?
- ◯ Female
- ◯ Male
- ◯ Prefer not to say

What is your gender?
- ◯ Female
- ◯ Male
- ◯ Gender diverse
- ◯ Prefer not to say

What is your sexual orientation?
- ◯ Heterosexual
- ◯ Heterosexual with homosexuality to some extent
- ◯ Bisexual
- ◯ Homosexual with heterosexuality to some extent
- ◯ Homosexual
- ◯ Asexual
- ◯ Unsure
- ◯ Prefer not to answer

Are you currently in a long term relationship with a partner?
- ◯ Yes - I have been with my current partner for at least 1 year
- ◯ Yes - I have been with my current partner for less than a year
- ◯ No - I do not have a partner currently
- ◯ Prefer not to say

In general, how satisfied are you with your relationship?
- ◯ Not at all satisfied
- ◯ Not very satisfied
- ◯ Somewhat satisfied
- ◯ Rather satisfied
- ◯ Very satisfied

In general, how satisfied are you with your sexual life?
- ◯ Not at all satisfied
- ◯ Not very satisfied
- ◯ Somewhat satisfied
- ◯ Rather satisfied
- ◯ Very satisfied

What is your current marital status?	◯ Married, civil union, de facto<br>◯ Separated/divorced/widowed<br>◯ Never married (single)<br>◯ Prefer not to say
What is your highest level of education, that you have either completed or are currently studying?	◯ Primary school/Elementary school<br>◯ Intermediate school/Middle school<br>◯ High school<br>◯ Trade or technical qualification<br>◯ Diploma or certificate<br>◯ University/college (undergraduate)<br>◯ University/college (postgraduate)
What is your employment status?	◯ Full-time: Paid employment for >=30 hrs a week<br>◯ Part-time: Paid employment for 1 to less than 30 hrs a week<br>◯ Not in Paid Employment OR Paid employment for less than 1 hour per week<br>◯ Retired

Next we will ask you some questions about religion.

How important is religion in your life?

- ◯ Not at all important
- ◯ Not too important
- ◯ Somewhat important
- ◯ Very important

What religious family do you belong to or identify most closely to?

- ◯ Catholic (including Roman Catholic and Orthodox)
- ◯ Protestant (e.g. Anglican, Orthodox, Baptist, Lutheran, United Church of Canada)
- ◯ Christian Orthodox
- ◯ Jewish
- ◯ Muslim
- ◯ Sikh
- ◯ Hindu
- ◯ Buddhist
- ◯ Atheist (do not believe in God)
- ◯ Agnostic (believe that the existence of God is unknowable)
- ◯ Other
- ◯ Prefer not to say

If other religion, please specify:

____

In the past 12 months, how often did you attend religious or worship services, not including weddings and funerals?

- ◯ Never
- ◯ Seldom
- ◯ A few times a year
- ◯ Once or twice a month
- ◯ Once a week
- ◯ More than once a week

Please tick the response that is closest to how you have been feeling in the past TWO WEEKS.

Don't take too long over your replies: your immediate response is best.

I feel tense or 'wound up':
- ◯ Most of the time
- ◯ A lot of the time
- ◯ From time to time, occasionally
- ◯ Not at all

I still enjoy the things I used to enjoy:
- ◯ Definitely as much
- ◯ Not quite so much
- ◯ Only a little
- ◯ Hardly at all

I get a sort of frightened feeling as if something awful is about to happen:
- ◯ Very definitely and quite badly
- ◯ Yes, but not too badly
- ◯ A little, but it doesn't worry me
- ◯ Not at all

I can laugh and see the funny side of things:
- ◯ As much as I always could
- ◯ Not quite so much now
- ◯ Definitely not so much now
- ◯ Not at all

Worrying thoughts go through my mind:
- ◯ A great deal of the time
- ◯ A lot of the time
- ◯ From time to time, but not too often
- ◯ Only occasionally

I feel cheerful:
- ◯ Not at all
- ◯ Not often
- ◯ Sometimes
- ◯ Most of the time

I can sit at ease and feel relaxed:
- ◯ Definitely
- ◯ Usually
- ◯ Not often
- ◯ Not at all

I feel as if I am slowed down:
- ◯ Nearly all the time
- ◯ Very often
- ◯ Sometimes
- ◯ Not at all

I get a sort of frightened feeling like 'butterflies' in my stomach:
- ◯ Not at all
- ◯ Occasionally
- ◯ Quite often
- ◯ Very often

I have lost interest in my appearance:
- ◯ Definitely
- ◯ I don't take as much care as I should
- ◯ I may not take quite as much care
- ◯ I take just as much care as ever

I feel restless as if I have to be on the move:
- ◯ Very much indeed
- ◯ Quite a lot
- ◯ Not very much
- ◯ Not at all

I look forward with enjoyment to things:	◯ As much as I ever did<br>◯ Rather less than I used to<br>◯ Definitely less than I used to<br>◯ Hardly at all
I get sudden feelings of panic:	◯ Very often indeed<br>◯ Quite often<br>◯ Not very often<br>◯ Not at all
I can enjoy a good book or radio or TV program:	◯ Often<br>◯ Sometimes<br>◯ Not often<br>◯ Very seldom

Please indicate how much you agree with the following statements, on a scale of 1 (completely disagree) to 5 (completely agree).

In the past TWO WEEKS:

	1 - No that is not true	2	3	4	5 - Yes that is true
When I have been doing something, I can keep my thoughts on it.	◯	◯	◯	◯	◯
I have been able to concentrate well.	◯	◯	◯	◯	◯
My thoughts have easily wandered.	◯	◯	◯	◯	◯
It has taken a lot of effort to concentrate on things.	◯	◯	◯	◯	◯

Please answer the following questions as either 'Yes' or 'No'.

	Yes	No
Do you smile at people every time you meet them?	○	○
Do you always practise what you preach to people?	○	○
If you say to people that you will do something, do you always keep your promise no matter how inconvenient it might be?	○	○
Would you ever lie to people?	○	○

Please indicate how much you agree with each of the following statements.

	Not at all	Slightly agree	Moderately agree	Strongly agree
I feel guilty, even though I do not know what it is caused by.	◯	◯	◯	◯
I experience moments when I cannot even look at myself.	◯	◯	◯	◯
There are moments when I would rather sink without trace.	◯	◯	◯	◯
When I do something wrong, I feel an exaggerated feeling of guilt.	◯	◯	◯	◯
I feel the need to explain or apologize for the reasons for my actions.	◯	◯	◯	◯
I am losing the hope that I will ever be a good person.	◯	◯	◯	◯
I blame myself for things that other people do not mind.	◯	◯	◯	◯
If I do anything wrong, I have to think about it all the time.	◯	◯	◯	◯

Please indicate how often each of the statements below is descriptive of you.

	I often feel this way	I sometimes feel this way	I rarely feel this way	I never feel this way
I lack companionship.	○	○	○	○
There is no one I can turn to.	○	○	○	○
I am an outgoing person.	○	○	○	○
I feel left out.	○	○	○	○
I feel isolated from others.	○	○	○	○
I can find companionship when I want it.	○	○	○	○
I am unhappy being so withdrawn.	○	○	○	○
People are around me but not with me.	○	○	○	○

Next we will ask you some questions about your drug and alcohol use. Your responses are entirely confidential and will not be shared with anyone.

In the past 90 DAYS, how often have you used the following substances?

	Never	Once or twice	Monthly	Weekly	Daily or almost daily
Alcohol	○	○	○	○	○
Cannabis (marijuana, pot, grass, hash, etc.)	○	○	○	○	○
Cocaine (coke, crack, etc.)	○	○	○	○	○
Prescription stimulants (Ritalin, Concerta, Dexedrine, Adderall, diet pills, etc.)	○	○	○	○	○
Methamphetamine (speed, crystal meth, ice, etc.	○	○	○	○	○
Inhalants (nitrous oxide, glue, gas, paint thinner, etc.)	○	○	○	○	○
Sedatives or sleeping pills (Valium, Serepax, Ativan, Librium, Xanax, Rohypnol, GHB, etc.)	○	○	○	○	○
Hallucinogens (LSD, acid, mushrooms, PCP, Special K, ecstasy, etc.)	○	○	○	○	○
Street opioids (heroin, opium, etc.)	○	○	○	○	○
Prescription opiods (fentanyl, oxycodone [OxyContin, Percocet], hydrocodone [Vicodin], methadone, buprenorphine, etc.)	○	○	○	○	○
Other	○	○	○	○	○

If 'other', please specify the substance: __________________________________

In the following sections, you will be asked questions about your sexual behavior. Please answer as honestly as you can. Your responses will be completely confidential.

Pornography is defined as material (text, audio, video, etc) that:
(i) creates or elicits sexual feelings or thoughts and
(ii) contains explicit exposure or descriptions of sexual acts involving the genitals, such as vaginal or anal intercourse, oral sex, or masturbation.

In the past 30 DAYS, on average how often have you:

	Never	Less than once a week	1-2 times per week	3-4 times per week	5-6 times per week	Once or twice a day	More than twice a day
Masturbated while using pornography?	◯	◯	◯	◯	◯	◯	◯
Masturbated WITHOUT using pornography?	◯	◯	◯	◯	◯	◯	◯
Had sexual intercourse with a partner?	◯	◯	◯	◯	◯	◯	◯

Are you currently an ACTIVE member of any online groups OR in-person groups to help you reduce your pornography use or masturbation frequency?

E.g., this may include groups such as the r/NoFap subreddit on reddit.com, or Sex Addicts Anonymous.

◯ Yes
◯ No

Please tick any of the following groups or online forums that you are currently an ACTIVE member of:

☐ r/nofap, r/nofapteens or r/nofapchristians on reddit.com, OR the nofap.com website
☐ r/pornfree or r/pornfreewomen on reddit.com
☐ Reboot Nation - rebootnation.org
☐ Your Brain Rebalanced - yourbrainrebalanced.com
☐ Fight The New Drug (FTND) - fightthenewdrug.org
☐ Your Brain On Porn (YBOP) - yourbrainonporn.com
☐ SMART Recovery - e.g. smartrecovery.org
☐ Sexaholics Anonymous
☐ Other

If other, please state the name of the group or forum that you are currently an ACTIVE member of:

____

Are you currently using any mobile apps or software to help you reduce your pornography use or masturbation frequency?

◯ Yes
◯ No

What features of this app/software do you use? You may choose multiple answers.<br><br>I am using the app/software:	☐ To block sexual content on the internet or my device<br>☐ To access tutorials/coaching sessions to help me reduce porn/masturbation<br>☐ To keep track of my 'streak length' (i.e. how long since I last used porn/masturbated)<br>☐ To connect with an 'accountability buddy', who checks my progress reducing porn/masturbation<br>☐ To keep a diary/journal of my progress<br>☐ To keep track of my mental health over time (e.g. tracking mood, anxiety, or stress levels on a daily basis)<br>☐ To keep track of my financial savings from not using pornography<br>☐ To distract me from using pornography or masturbating (e.g. through playing games, meditating, or using a 'panic button')<br>☐ Other
If other, please describe why you are using the app/software:	__________________________________

Please think back to the past SIX MONTHS and indicate how much the following statements apply to you.

	Never	Rarely	Occasionally	Sometimes	Often	Very often	All the time
I felt that porn is an important part of my life.	○	○	○	○	○	○	○
I released my tension by watching porn.	○	○	○	○	○	○	○
I neglected other leisure activities as a result of watching porn.	○	○	○	○	○	○	○
I felt that I had to watch more and more porn for satisfaction.	○	○	○	○	○	○	○
When I vowed not to watch porn anymore, I could only do it for a short period of time.	○	○	○	○	○	○	○
I became stressed when something prevented me from watching porn.	○	○	○	○	○	○	○

How much do you agree with the following statements?

	Not at all			Somewhat			Very strongly
I believe that masturbating WITH pornography is morally wrong.	○	○	○	○	○	○	○
I believe that masturbating WITHOUT pornography is morally wrong.	○	○	○	○	○	○	○
I believe that using pornography WITHOUT masturbating is morally wrong.	○	○	○	○	○	○	○
Often I have felt strong discomfort because my sexual fantasies, thoughts and behaviors were inconsistent with my moral and/or religious beliefs.	○	○	○	○	○	○	○

Thank you for completing this survey. We will send you an email shortly with further instructions.

To show our appreciation for your time, If you complete at least 60% of the surveys in the next part of this study (as well as the final survey), we will send you a $50 NZD gift voucher.

Would you prefer this voucher to be an Amazon gift card or a Westfield shopping voucher?

○ Amazon gift card
○ Westfield shopping voucher
