# Supplementary material for: Quantifying Opponent Process Dynamics in Pornography Use and Masturbation: An Exploratory Ecological Momentary Assessment Study
Source: Arch Sex Behav. 2025 Nov 21;54(9):3313–34. doi: 10.1007/s10508-025-03287-z (PMC12675693; doi:10.1007/s10508-025-03287-z)
Supplement: Supplementary file 3 — Supplementary file3 (PDF 152 KB) [file 10508_2025_3287_MOESM3_ESM.pdf]

**<u>SEMA3 surveys - data dictionary</u>**		
**Survey: "Complete this survey EACH TIME you use porn, masturbate, or have sex."**		
**(Ad-hoc survey)**		
Variable name	***Description***	***Attributes***
TYPE_PMOS	What type of sexual activity was performed? (Scroll down to see all the options.)	1 = Used porn WITHOUT masturbation, NO orgasm<br>2 = Used porn AND masturbated but WITHOUT orgasm<br>3 = Used porn AND masturbated WITH orgasm<br>4 = Masturbated WITHOUT porn, NO orgasm<br>5 = Masturbated WITH orgasm but WITHOUT porn<br>6 = Had sex WITH a partner but WITHOUT orgasm<br>7 = Had sex WITH a partner WITH orgasm
PORN_TRIGGERS	What were the events or triggers that led to you using pornography?	Text
LENGTH_PORN	How long was your session of pornography use, in minutes? (Try to be accurate to the nearest minute, e.g. '12')	Number
MOOD_PORN	On a scale of 0-10, what was the HIGHEST LEVEL of happiness you experienced while using porn? 0 = extremely unhappy, 5 = neutral, 10 = extremely happy.	Integer scale, 0-10

STRENGTH_ORGASM	On a scale of 0-10, how strong was your orgasm? 0 = no orgasm, 5 = moderate orgasm, 10 = very strong orgasm.	Integer scale, 0-10
LENGTH_SEX	How long was your session of sexual intercourse, in minutes? (Try to be accurate to the nearest minute, e.g. '12')	Number
MOOD_SEX	On a scale of 0-10, what was the HIGHEST LEVEL of happiness you experienced while having sex? 0 = extremely unhappy, 5 = neutral, 10 = extremely happy.	Integer scale, 0-10
MAST_TRIGGERS	What were the events or triggers that led to you masturbating?	Text
LENGTH_MAST	How long was your session of masturbation, in minutes? (Try to be accurate to the nearest minute, e.g. '12')	Number
MOOD_MAST	On a scale of 0-10, what was the HIGHEST LEVEL of happiness you experienced while masturbating? 0 = extremely unhappy, 5 = neutral, 10 = extremely happy.	Integer scale, 0-10
TODAY_PMOS	Are you recording sexual activity that happened today, or on a previous day?	0 = Today<br>1 = On a previous day
DATE_PMOS	How many days ago was your last instance of sexual activity? (E.g. if it was 3 days ago, enter '3')	Number
TIME_PMOS	At what time did this sexual activity end? (Try to be accurate to the nearest five minutes if possible, e.g., "21:15")	Time (HH:mm)
MOOD_PMOS	On a scale of 0-10, what is your CURRENT mood? 0 = extremely unhappy, 5 = neutral, 10 = extremely happy.	Integer scale, 0-10
ANXIETY_PMOS	On a scale of 0-10, how anxious do you CURRENTLY feel? 0 = no anxiety, 5 = moderate anxiety, 10 = extremely high anxiety.	Integer scale, 0-10
GUILT_PMOS	On a scale of 0-10, how guilty do you feel RIGHT NOW about your sexual behaviors (sex/porn/masturbation)? 0 = no guilt, 5 = some guilt, 10 = very guilty.	Integer scale, 0-10

LONELINESS_PMOS	On a scale of 0-10, how lonely do you CURRENTLY feel? 0 = no loneliness, 5 = moderate loneliness, 10 = extremely lonely.	Integer scale, 0-10
THINKING_PMOS	On a scale of 0-10, how much difficulty are you having with thinking clearly RIGHT NOW? 0 = no difficulty, 5 = moderate difficulty, 10 = extreme difficulty.	Integer scale, 0-10
CONNECTED_PMOS	AT THIS MOMENT, how emotionally connected do you feel to your loved ones? 0 = not connected at all, 5 = moderately connected, 10 = very connected.	Integer scale, 0-10
SHAME_PMOS	On a scale of 0-10, how much shame are you feeling RIGHT NOW? 0 = no shame, 5 = moderate shame, 10 = extremely ashamed.	Integer scale, 0-10
CRAVING_PORN_PMOS	On a scale of 0-10, what is your CURRENT level of craving for porn and/or masturbation? 0 = no craving, 5 = moderate craving, 10 = extremely strong craving.	Integer scale, 0-10
CRAVING_SEX_PMOS	On a scale of 0-10, what is your CURRENT level of craving for sex with a partner? 0 = no craving, 5 = moderate craving, 10 = extremely strong craving.	Integer scale, 0-10
**Survey: "Complete this survey NOW!"**		
**(Schedule-contingent survey)**		
Variable name	***Description***	***Attributes***
MOOD	On a scale of 0-10, what is your CURRENT mood? 0 = extremely unhappy, 5 = neutral, 10 = extremely happy.	Integer scale, 0-10
ANXIETY	On a scale of 0-10, how anxious do you CURRENTLY feel? 0 = no anxiety, 5 = moderate anxiety, 10 = extremely high anxiety.	Integer scale, 0-10

GUILT	On a scale of 0-10, how guilty do you feel RIGHT NOW about your sexual behaviors (sex/porn/masturbation)? 0 = no guilt, 5 = some guilt, 10 = very guilty.	Integer scale, 0-10
LONELINESS	On a scale of 0-10, how lonely do you CURRENTLY feel? 0 = no loneliness, 5 = moderate loneliness, 10 = extremely lonely.	Integer scale, 0-10
THINKING	On a scale of 0-10, how much difficulty are you having with thinking clearly RIGHT NOW? 0 = no difficulty, 5 = moderate difficulty, 10 = extreme difficulty.	Integer scale, 0-10
CONNECTED	AT THIS MOMENT, how emotionally connected do you feel to your loved ones? 0 = not connected at all, 5 = moderately connected, 10 = very connected.	Integer scale, 0-10
SHAME	On a scale of 0-10, how much shame are you feeling RIGHT NOW? 0 = no shame, 5 = moderate shame, 10 = extremely ashamed.	Integer scale, 0-10
CRAVING_PORN	On a scale of 0-10, what is your CURRENT level of craving for porn and/or masturbation? 0 = no craving, 5 = moderate craving, 10 = extremely strong craving.	Integer scale, 0-10
CRAVING_SEX	On a scale of 0-10, what is your CURRENT level of craving for sex with a partner? 0 = no craving, 5 = moderate craving, 10 = extremely strong craving.	Integer scale, 0-10
